# Pyrethroid-Degrading Microorganisms and Their Potential for the Bioremediation of Contaminated Soils: A Review

**DOI:** 10.3389/fmicb.2016.01463

**Published:** 2016-09-15

**Authors:** Mariusz Cycoń, Zofia Piotrowska-Seget

**Affiliations:** ^1^Department of Microbiology and Virology, School of Pharmacy with the Division of Laboratory Medicine, Medical University of SilesiaSosnowiec, Poland; ^2^Department of Microbiology, University of SilesiaKatowice, Poland

**Keywords:** pyrethroids, microorganisms, biodegradation pathways, bioremediation, enzymes, soil

## Abstract

Pyrethroid insecticides have been used to control pests in agriculture, forestry, horticulture, public health and for indoor home use for more than 20 years. Because pyrethroids were considered to be a safer alternative to organophosphate pesticides (OPs), their applications significantly increased when the use of OPs was banned or limited. Although, pyrethroids have agricultural benefits, their widespread and continuous use is a major problem as they pollute the terrestrial and aquatic environments and affect non-target organisms. Since pyrethroids are not degraded immediately after application and because their residues are detected in soils, there is an urgent need to remediate pyrethroid-polluted environments. Various remediation technologies have been developed for this purpose; however, bioremediation, which involves bioaugmentation and/or biostimulation and is a cost-effective and eco-friendly approach, has emerged as the most advantageous method for cleaning-up pesticide-contaminated soils. This review presents an overview of the microorganisms that have been isolated from pyrethroid-polluted sites, characterized and applied for the degradation of pyrethroids in liquid and soil media. The paper is focused on the microbial degradation of the pyrethroids that have been most commonly used for many years such as allethrin, bifenthrin, cyfluthrin, cyhalothrin, cypermethrin, deltamethrin, fenpropathrin, fenvalerate, and permethrin. Special attention is given to the bacterial strains from the genera *Achromobacter, Acidomonas, Bacillus, Brevibacterium, Catellibacterium, Clostridium, Lysinibacillus, Micrococcus, Ochrobactrum, Pseudomonas, Serratia, Sphingobium, Streptomyces*, and the fungal strains from the genera *Aspergillus, Candida, Cladosporium*, and *Trichoderma*, which are characterized by their ability to degrade various pyrethroids. Moreover, the current knowledge on the degradation pathways of pyrethroids, the enzymes that are involved in the cleavage of pesticide molecules, the factors/conditions that influence the survival of strains that are introduced into soil and the rate of the removal of pyrethroids are also discussed. This knowledge may be useful to optimize the environmental conditions of bioremediation and may be crucial for the effective removal of pyrethroids from polluted soils.

## Introduction

Synthetic pyrethroids (SPs) are the chemical analogs of pyrethrins, which are compounds that are present in the flowers of *Chrysanthemum cinerariaefolium*. Pyrethrins have been recognized as active insecticide compounds; however, due to their rapid degradation in the environment, they have never been used for plant protection on a large scale in agriculture (Laskowski, 2002; Palmquist et al., 2012). Modifications of the molecular structure of pyrethrins resulted in the synthesis of two generations of SPs. The first was developed in the 1960s and involved several pyrethrin derivatives such as bioallethrin, tetramethrin, resmethrin, and bioresmethrin. Although, these pesticides were more active than natural pyrethrum, they were unstable in sunlight, which limited their use. In the 1970s a second generation of SPs including permethrin, cypermethrin and deltamethrin was developed. In the subsequent years, other insecticides such as fenvalerate, lambda-cyhalothrin and beta-cyfluthrin were synthesized (Kidd and James, 1991; Katsuda, 1999). Compared to natural pyrethrins, synthetic pyrethroids are more stable in direct sunlight and are significantly more effective against a wide range of insects. These properties made them much more suitable for use in agriculture (Laskowski, 2002). Beginning in 2000 when the use of organophosphorus pesticides decreased, the market for pyrethroid pesticides increased significantly. Nowadays, pyrethroids contribute more than 25% of the world's total pesticide market (Laffin et al., 2010; Pérez et al., 2010; Chen et al., 2011a).

Pyrethroids are insecticides that have a high biological activity and are used all over the world to control pest insects in agriculture, public and commercial buildings, animal facilities, greenhouses, and veterinary facilities (Katsuda, 1999). Pyrethroids are also the most common active ingredients in commercially available insect sprays and are the domain pesticide for malaria control. The insecticidal potency of pyrethroids is connected with the induction of a toxic effect in the cells of the nervous system of insects (Burr and Ray, 2004). By permitting a flux of sodium ions, pyrethroids alter the activity of the sodium channels that are responsible for the signal transmissions of nerve impulses. When pyrethroids bind to target channel proteins, they disrupt the proper function of the nervous cells thus leading to paralysis and the eventual death of insects (Burr and Ray, 2004; Davies et al., 2007; Hintzen et al., 2009).

Pyrethroids differ from many other pesticides in that they contain one to three chiral centers; their chirality may arise from the acid moiety, the alcohol moiety or both. A pyrethroid compound, therefore, consists of two to eight isomers. The isomers of a chiral compound often differ from each other in their biological properties. Isomer selectivity has been widely observed for the isomers of a pyrethroid compound in insecticidal activity (Wauchope et al., 1992; Katsuda, 1999). Recently, studies have shown that the biodegradation of pyrethroids also exhibits significant isomer selectivity. Based on their toxicological and physical properties, pyrethroids are categorized into two separate classes—type I and type II. Type I pyrethroids, which include allethrin, bifenthrin, d-phenothrin, permethrin, resmethrin, and tetramethrin, do not have a cyano group. Conversely, the insecticides that represent Type II such as cyhalothrin, cypermethrin, cyfluthrin, deltamethrin, fenvalerate, fluvalinate, and lambda-cyhalothrin have a cyano group in their structure (Laskowski, 2002). Due to their complex chemical structure, pyrethroids are composed of two, four or eight isomers and their commercial products may contain a mixture of these various isomers. The production of individual pyrethroids that have varying isomeric ratios may be the reason for the variations in the toxicity of the same compound (Delgado-Moreno et al., 2011). In addition, pyrethroids represent highly hydrophobic compounds that are characterized by their low water solubility, which ranges from insolubility to a value of 0.1 mg/L and high octanol-water partition coefficients (Wauchope et al., 1992; Laskowski, 2002; Tomlin, 2003).

Although, pyrethroids are considered to be safer than other insecticides, the common and extensive use of these compounds in a wide variety of fields has resulted in widespread contamination of the environment that is of ecological concern. The results of many studies have revealed that SPs may negatively affect non-target organisms such as fish and aquatic insects (Wendt-Rasch et al., 2003; Antwi and Reddy, 2015), beetles (Desneux et al., 2007), bees (Decourtye et al., 2005), parasitic wasps (Longley and Jepson, 1996) and microorganisms (Widenfalk et al., 2004; Cycoń et al., 2006; Das et al., 2016). It is thought that some pyrethroids may be responsible for disruptions of the endocrine system, suppression of the immune system, reproductive damage and increased chances of cancer in humans (ATSDR (Agency for Toxic Substances and Disease Registry), 2003; Zhang et al., 2010).

To reduce the environmental and public health risks associated with pyrethroid use, it is necessary to develop rapid and effective methods to remove or minimize the concentrations of insecticides in the environment. Among the variety of methods that are used for the remediation of contaminated environments, the biological approach, which is based on the catabolic activity of pesticide-degrading bacteria, seems to be the most promising and effective strategy (Chen et al., 2012c; Zhao et al., 2013; Cycoń et al., 2014; Akbar et al., 2015a). The results of many studies have proven that pyrethroids can be successfully eliminated from media and soils by diverse microorganisms that belong to different taxonomic groups. In this review we focus on the microbial-mediated degradation of the following synthetic pyrethroids: allethrin, bifenthrin, cypermethrin, cyfluthrin, λ-cyhalothrin, deltamethrin, fenpropathrin, fenvalerate, and permethrin whose basic descriptions are presented in Table 1.

**Table 1:** **Basic description of pyrethroids**.

**Pyrethroid**	**Chemical name**	**Chemical formula**	**MW (g/mol)**	**Chemical structure**
Allethrin	(2-Methyl-4-oxo-3-prop-2-enylcyclopent-2-en-1-yl) 2,2-dimethyl-3-(2-methylprop-1-enyl)cyclopropane-1-carboxylate	C_19_H_26_O_3_	302.41	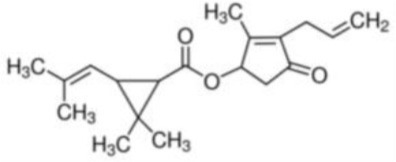
Bifenthrin	(2-Methyl-3-phenylphenyl)methyl (1*R*,3*R*)-3-[(*Z*)-2-chloro-3,3,3-trifluoroprop-1-enyl]-2,2-dimethylcyclopropane-1-carboxylate	C_23_H_22_ClF_3_O_2_	422.87	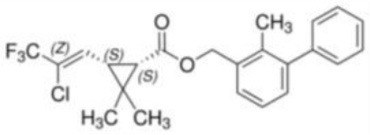
Cyfluthrin	[(*R*)-Cyano-[4-fluoro-3-(phenoxy)phenyl]methyl] (1*R*,3*R*)-3-(2,2-dichloroethenyl)-2,2-dimethylcyclopropane-1-carboxylate	C_22_H_18_Cl_2_FNO_3_	434.29	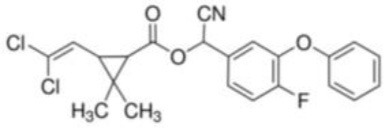
Cyhalothrin	3-(2-Chloro-3,3,3-trifluoro-1-propenyl)-2,2-dimethyl-cyano(3-phenoxyphenyl)methyl cyclopropanecarboxylate	C_23_H_19_ClF_3_NO_3_	449.85	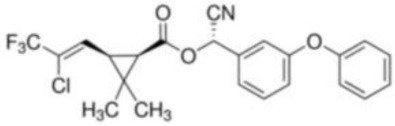
Cypermethrin	[Cyano-(3-phenoxyphenyl)methyl]3-(2,2-dichloroethenyl)-2,2-dimethylcyclopropane-1-carboxylate	C_22_H_19_Cl_2_NO_3_	416.30	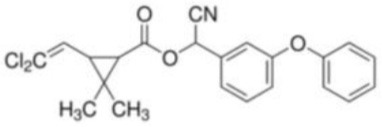
Deltamethrin	[(*S*)-Cyano-(3-phenoxyphenyl)-methyl] (1*R*,3R)-3-(2,2-dibromoethenyl)-2,2-dimethyl-cyclopropane-1-carboxylate	C_221_H_19_Br_2_NO_3_	505.21	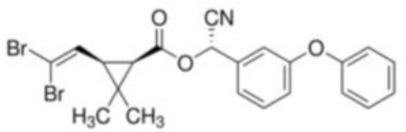
Fenpropathrin	[Cyano-(3-phenoxyphenyl)methyl] 2,2,3,3-tetramethylcyclopropane-1-carboxylate	C_22_H_23_NO_3_	349.43	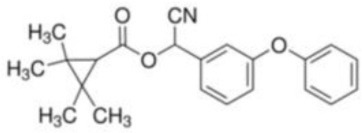
Fenvalerate	(*RS*)-alpha-Cyano-3-phenoxybenzyl (*RS*)-2-(4-chlorophenyl)-3-methylbutyrate	C_25_H_22_ClNO_3_	419.91	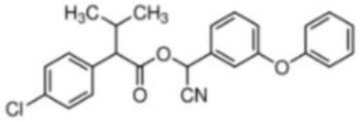
Permethrin	(±)-3-Phenoxybenzyl 3-(2,2-dichlorovinyl)-2,2-dimethylcyclopropanecarboxylate	C_21_H_20_Cl_2_O_3_	391.29	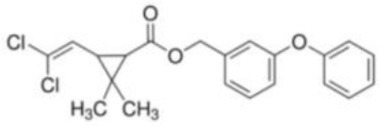

### Pyrethroids in soil environment

The wide use and repeated applications of SPs has led to their permanent occurrence in some environments, especially in soils where they can be toxic to target and non-target organisms (Oudou and Hansen, 2002; Hintzen et al., 2009; Meyer et al., 2013; Xu et al., 2015). Due to their highly hydrophobic properties, pyrethroids strongly bind to soil particles and organic matter, which allows them to leach into the groundwater and to form residues of these compounds, thereby adversely affecting the ecosystem (Jin and Webster, 1998; Oudou and Hansen, 2002; Singh and Singh, 2004; Gu et al., 2008; Xu et al., 2015). The results of many studies have shown that pyrethroids can have many harmful effects on soil biology that involve quantitative and qualitative changes in the soil microflora, changes in the activity of enzymes and alterations in the nitrogen balance of the soil (inhibition of N_2_ fixing and nitrifying microorganisms as well as interference with ammonification). The direct and indirect impact of pyrethroids on the microbiological aspects of soil then affect plant growth and soil fertility (Cycoń et al., 2006; Boucard et al., 2008; Zhang et al., 2009; Muñoz-Leoz et al., 2013; Filimon et al., 2015; Tejada et al., 2015; Das et al., 2016).

Pyrethroids undergo many different pathways once they enter the soil environment, including transformation/degradation, sorption-desorption, volatilization, uptake by plants, runoff into surface waters, and transport into groundwater. Transformation or degradation is one of the key processes that governs the environmental fate and transport of pyrethroids, which also comprises different processes including abiotic degradation (e.g., oxidation, hydrolysis and photolysis) and biodegradation. During these processes, pyrethroids are transformed into a degradation product or completely mineralized. However, the structure of a pyrethroid molecule determines its inherent biodegradation (Gupta and Gajbhiye, 2002; Gu et al., 2008; Muñoz-Leoz et al., 2009; Yuanfan et al., 2010; Fenlon et al., 2011; Ismail et al., 2012; Zhao et al., 2013; Xu et al., 2015; Zhang et al., 2016). In the environmental degradation of pyrethroids ester cleavage is a major process that results most often in the production of cyclopropane acid, 3-phenoxybenzyl alcohol, 3-phenoxybenzaldehyde (3-PBA) or 3-phenoxybenzoic acid. 3-Phenoxybenzyl alcohol also often is an intermediate in the photocatabolism of pyrethroids, which can then undergo oxidation to form the corresponding carboxylic acid (Tyler et al., 2000; Wang et al., 2011; Xiao et al., 2015). Among the mentioned metabolites, 3-PBA often accumulates in soil and further degradation of pyrethroids and 3-PBA may be limited or inhibited due to its strong antimicrobial activity (Xu et al., 2007; Xia et al., 2008; Chen et al., 2011d).

The abiotic degradation of pyrethroids is substantial in many cases; however, this process is slow under anaerobic or sterile conditions. As has been shown in many studies, the half-life or DT50 values of pyrethroids are much lower in soils with autochthonous microorganisms compared to those that are obtained for sterilized soils, which indicates the important role of microorganisms in the transformation of these pesticides. Depending on the type of soil and the initial concentration, the half-life values of bifenthrin, cyfluthrin, cypermethrin, deltamethrin, fenpropathrin, fenvalerate, and permethrin in non-sterilized soils have been estimated to be 12.4–1410, 7.8–54.6, 17.1–52.1, 8.3–105.3, 37.1, 17.7–41.3, and 5–55 days, respectively (Table 2).

**Table 2:** **Degradation of selected pyrethroids in soils of different characteristics under laboratory conditions**.

**Pyrethroid**	**Type of soil**	**Main properties of soil**	**Conditions**	**Dose (mg/kg)**	***T*_1/2_/DT_50_(comments)**	**References**
Bifenthrin	Red brown earth	Sand 59.1%, silt 28.3%, clay 10.5%, pH 7.1, OM 1.2%	25°C, 60% WHC	100	*T*_1/2_ 1332 days (b.d.)	Baskaran et al., 1999
	Quarry sand	Sand 80.2%, pH 9.2, OM 0.1%	25°C, 60% WHC	100	*T*_1/2_ 1410 days (b.d.)	
	Sandy loam	Sand 65%, silt 28%, clay 7%, pH 6.9, OM 10.5 g/kg	32°C, 40% WHC	50	*T*_1/2_ 130.7 days (a.d.), *t*_1/2_ 78.8 days (b.d.)	Chen et al., 2012d
	Light clay	Sand 21.4%, silt 28.6%, clay 40%, pH 5.5, OM 3.8%	25°C, 50% WHC	1	*T*_1/2_ 12.4 days (b.d.)	Kim and Choi, 1992
	Clay loam	Sand 41.9%, silt 39.9%, clay 18.2%, pH 6.7, OM 1.1%	25°C, 50% WHC	1	*T*_1/2_ 85.1 days (b.d.)	
Cyfluthrin	Light clay	Sand 21.4%, silt 28.6%, clay 40%, pH 5.5, OM 3.8%	25°C, 50% WHC	1	*T*_1/2_ 32.2 days (b.d.)	
	Clay loam	Sand 77.7%, silt 17.5%, clay 5%, pH 6.7, OM 1.1%	25°C, 50% WHC	1	*T*_1/2_ 54.6 days (b.d.)	
	Sandy loam	Sand 41.9%, silt 39.9%, clay 18.2%, pH 7.7, OM 0.5%	25°C,	0.1, 1 and 10	*T*_1/2_ 7.8–41.8 days (b.d.)	Gupta and Gajbhiye, 2002
Cypermethrin	No data	pH 7.9, OM 54.9 mg/g	22°C, 40% WHC	20	*T*_1/2_ 52.1 days (b.d.)	Liu et al., 2014
	No data	pH 6.7, OM 11.3 g/kg	30°C	50	*T*_1/2_ 40.5 days (a.d.), *t*_1/2_ 21.6 days (b.d.)	Zhao et al., 2013
	No data	pH 4.77, OM 1.12%	30°C, 60% WHC	10	*T*_1/2_ 105.8 days (a.d.), *t*_1/2_ 49.4 days (b.d.)	Xu et al., 2015
	No data	No data	30°C, 40% WHC	200	*T*_1/2_ 101.9 days (a.d.), *t*_1/2_ 48.5 days (b.d.)	Akbar et al., 2015b
	Ortho red	Sand 7%, silt 59%, clay 34%, pH 6.1, OM 24.5 mg/kg	25°C, 25% WHC	2	*T*_1/2_ 19.5 days (b.d.)	Gu et al., 2008
	Yellow red	Sand 15%, silt 57%, clay 28%, pH 6.9, OM 30.8 mg/kg	25°C, 25% WHC	2	*T*_1/2_ 76.2 days (a.d.), *t*_1/2_ 17.1 days (b.d.)	
	Brown red	Sand 13%, silt 58%, clay 29%, pH 7.1, OM 33.6 mg/kg	25°C, 25% WHC	2	*T*_1/2_ 14.4 days (b.d.)	
	Sandy loam	Sand 65%, silt 28%, clay 7%, pH 6.9, OM 10.5 g/kg	28°C, 40% WHC	50	*T*_1/2_ 57.3 days (a.d.), *t*_1/2_ 24.1 days (b.d.)	Chen et al., 2012c
Deltamethrin	No data	pH 6.4, OM 28 g/kg	30°C, 30% WHC	10	*T*_1/2_ 12.8 days (a.d.), *t*_1/2_ 8.3 days (b.d.)	Zhang et al., 2016
	Sand	Sand 86%, silt 11%, clay 3%, pH 6.8, Corg 1.4%	30°C, 50% WHC	100	DT_50_ 234.5 days (a.d.), DT_50_ 71.9 days (b.d.)	Cycoń et al., 2014
	Sandy loam	Sand 91%, silt 6%, clay 3%, pH 6.5, Corg 1.1%	30°C, 50% WHC	100	DT_50_ 315.3 days (a.d.), DT_50_ 68.8 days (b.d.)	
	Silty loam	Sand 69%, silt 21%, clay 10%, pH 6.7, Corg 1.6%	30°C, 50% WHC	100	DT_50_ 433.8 days (a.d.), DT_50_ 86.4 days (b.d.)	
	Silt	Sand 18%, silt 76%, clay 6%, pH 6.8, Corg 1.4%	30°C, 50% WHC	100	DT_50_ 537.8 days (a.d.), DT_50_ 105.3 days (b.d.)	
Deltamethrin	Ortho red	Sand 7%, silt 59%, clay 34%, pH 6.1, OM 24.5 mg/kg	25°C, 25% WHC	2	*T*_1/2_ 20.3 days (b.d.)	Gu et al., 2008
	Yellow red	Sand 15%, silt 57%, clay 28%, pH 6.9, OM 30.8 mg/kg	25°C, 25% WHC	2	*T*_1/2_ 84.5 days (a.d.), *t*_1/2_ 18.4 days (b.d.)	
	Brown red	Sand 13%, silt 58%, clay 29%, pH 7.1, OM 33.6 mg/kg	25°C, 25% WHC	2	*T*_1/2_ 15.7 days (b.d.)	
	Silty loam	Sand 29.8%, silt 31.5%, clay 38.7%, pH 8.3, Corg 1.7 g/kg	20°C, 75% WHC	50	*T*_1/2_ 27.4 days (b.d.)	Muñoz-Leoz et al., 2009
	Silty loam	Sand 29.8%, silt 31.5%, clay 38.7%, pH 8.3, Corg 1.7 g/kg	20°C, 75% WHC	125	*T*_1/2_ 47.4 days (b.d.)	
	Silty loam	Sand 29.8%, silt 31.5%, clay 38.7%, pH 8.3, Corg 1.7 g/kg	20°C, 75% WHC	250	*T*_1/2_ 44.4 days (b.d.)	
Fenpropathrin	Sandy loam	Sand 65%, silt 28%, clay 7%, pH 6.9, OM 10.5 g/kg	30°C, 40% WHC	50	*T*_1/2_ 70.7 days (a.d.), *t*_1/2_ 37.1 days (b.d.)	Chen et al., 2014
	Sandy loam	Sand 66%, silt 11%, clay 16%, pH 6.6, OM 2.3%	30°C, 40% WHC	50	< 25% degradation (b.d.)	Yuanfan et al., 2010
Fenvalerate	Peat soil	Sand 3%, silt 1%, clay 96%, pH 4.4, OM 82.8%	30°C, 50% WHC, 35°C, 50% WHC	100	*T*_1/2_ 41.3 days (b.d.), *t*_1/2_ 28.7 days (b.d.)	Ismail and Maznah, 2005
	Sandy clay	Sand 52%, silt 10%, clay 38%, pH 5.2, OM 12.7%	30°C, 50% WHC, 35°C, 50% WHC	100	*T*_1/2_ 28.7 days (b.d.), *t*_1/2_ 20.3 days (b.d.)	
	Sandy clay loam	Sand 51%, silt 27%, clay 22%, pH 6.6, OM 3.6%	30°C, 50% WHC, 35°C, 50% WHC	100	*T*_1/2_ 23.8 days (b.d.), *t*_1/2_ 19.6 days (b.d.)	
	Ortho red	Sand 7%, silt 59%, clay 34%, pH 6.1, OM 24.5 mg/kg	25°C, 25% WHC	2	*T*_1/2_ 25.2 days (b.d.)	Gu et al., 2008
	Yellow red	Sand 15%, silt 57%, clay 28%, pH 6.9, OM 30.8 mg/kg	25°C, 25% WHC	2	*T*_1/2_ 92.4 days (a.d.), *t*_1/2_ 19.6 days (b.d.)	
	Brown red	Sand 13%, silt 58%, clay 29%, pH 7.1, OM 33.6 mg/kg	25°C, 25% WHC	2	*T*_1/2_ 17.7 days (b.d.)	
	Silty loam	Sand 36%, silt 58%, clay 6%, pH 6.7, OM 10.4 g/kg	30°C, 40% WHC	50	*T*_1/2_ 28.4 days (a.d.), *t*_1/2_ 19.2 days (b.d.)	Chen et al., 2011c
Permethrin	Mineral soil	pH 8.0-8.1	No data	1	94% removal after 8 weeks (b.d.)	Chapman et al., 1981
	Organic soil	pH 7.1-7.2	No data	1	84% removal after 8 weeks (b.d.)	
	Organic soil	pH 6.5-6.9	No data	1	No degradation (a.d.)	
	Mineral soil	pH 7.7-8.1	No data	1	No degradation (a.d.)	
	Sandy loam	pH 5.9; OM 1.0%	No data	1	*T*_1/2_ 55 days (cis-permethrin), *t*_1/2_ 5 days (trans-permethrin) (b.d.)	Jordan et al., 1982

OM, organic matter; WHC, water holding capacity; a.b., abiotic degradation; b.d., biotic degradation.

The degradation efficiency of pyrethroids depends not only on the catabolic activity of soil microorganisms but also to a large extent on the different properties of soil, i.e., soil texture, organic matter content, moisture, pH, and temperature (Gu et al., 2008; Chen et al., 2012d; Xu et al., 2015; Zhang et al., 2016). Detailed information on the degradation rates of SPs in soils of different characteristics are presented in Table 2. For example, Ismail et al. (2012) studied the effects of temperature, soil moisture content and soil type on the degradation of cypermethrin in two agricultural soils. The half-life of cypermethrin decreased from 5.9 to 3.2 weeks when the temperature was increased from 25 to 35°C. Results also showed that the half-life decreased from 6.6 to 2.5 weeks when soil moisture content increased from 40 to 60% (Ismail et al., 2012). In another study by Ismail and Maznah (2005), the degradation of fenvalerate was also affected by the temperature and the moisture content, the increases of which resulted in the decrease of half-life of the insecticide. Cycoń et al. (2014) revealed a correlation between the texture of soil and the organic matter content and the rate of the degradation of deltamethrin. Generally, a lower content of clay and organic matter in soil resulted in a higher degradation of deltamethrin. The higher ratio of deltamethrin dissipation that was observed in sandy soils that were characterized by a low content of organic matter and clay fraction was connected with the higher availability of the insecticide to bacteria compared to silty soil (Cycoń et al., 2014). Similarly, a correlation between the dissipation of cypermethrin, deltamethrin, and fenvalerate and soil organic matter content was observed by Gu et al. (2008). However, in contrast to results obtained by Cycoń et al. (2014), they found the highest dissipation of pyrethroids in soils that characterized by the highest organic matter content. These results showed that organic matter and clay content are the major factors that control the bioavailability of pyrethroids for microorganisms. The lipophilic properties of pyrethroids result in their strong tendency to bind various organic and non-organic soil components and these insecticides can persist in soils for a long period. However, their adsorption and desorption processes are associated with other soil parameters such as pH and water content (Oudou and Hansen, 2002; Gu et al., 2008; Muñoz-Leoz et al., 2009).

### Biodegradation of pyrethroids in liquid media

Contaminated sites are considered to be a good source for the isolation of pyrethroid-degrading microorganisms (Tables 3, 4). Using enrichment culture techniques, several bacterial and fungal species belonging to various genera have been isolated from pyrethroid-contaminated soils (Wang et al., 2011; Cycoń et al., 2014; Akbar et al., 2015a; Bhatt et al., 2016; Lee et al., 2016), sludge (Yuanfan et al., 2010; Chen et al., 2011a; Sundaram et al., 2013; Xiao et al., 2015; Tiwary and Dubey, 2016), or wastewater (Lin et al., 2011; Zhang S. et al., 2011; Chen et al., 2014). For the effective use of microorganisms in the remediation of pyrethroid-contaminated soils, it is extremely important to determine the potential of these microorganisms for the degradation of pyrethroids under optimal conditions in liquid media. Many studies have confirmed that several bacterial (Table 3) and fungal (Table 4) species are capable of degrading pyrethroids in liquid cultures. As is illustrated in these tables, microorganisms can degrade pyrethroids by either using them directly as a source of carbon (e.g., Paingankar et al., 2005; Chen et al., 2014; Cycoń et al., 2014; Liu et al., 2014; Akbar et al., 2015b) or co-metabolically (e.g., Saikia and Gopal, 2004; Chen et al., 2011a; Zhang S. et al., 2011; Lee et al., 2016). However, the rate of the biodegradation process in liquid media is influenced by many factors including temperature, pH, nutrients, pyrethroid concentration, inoculum size as well as the properties of the bacterial or fungal strains (Saikia and Gopal, 2004; Zhang et al., 2010; Zhao et al., 2013; Chen et al., 2015).

**Table 3:** **Pyrethroid-degrading bacteria isolated from contaminated sites and their degradation potential in liquid media**.

**Strain**	**Source**	**Degraded pyrethroid**	**Mode of action**	**Optimal conditions**	**Comments**	**References**
*Achromobacter* sp. (SM-2)	Soil and sewage sludge, UK	Permethrin	Co-metabolic	pH 7, 30°C	70–90% degradation (20 mg/L) in the presence of Tween 80 within 4 weeks (depended on the type of isomer)	Maloney et al., 1988
*Acidomonas* sp.	Soil, India	Allethrin	Catabolic	pH 7, 37°C	More than 70% of initial concentration (5 g/L) was degraded within 72 h	Paingankar et al., 2005
*Acinetobacter calcoaceticus* MCm5	Soil with history of pyrethroid application, Pakistan	Cypermethrin	Catabolic	pH 7, 30°C	84.7% of cypermethrin (100 mg/L) removal after 10 days	Akbar et al., 2015a
		Bifenthrin			About 78% of pesticide (100 mg/L) was degraded in 7 days	
		Cyhalothrin			Approximately 62% removal (100 mg/L) within 7 days	
		Deltamethrin			About 73% of pesticide (100 mg/L) was degraded in 7 days	
*Azoarcus indigens* HZ5	Activated sludge, China	Cypermethrin	Co-metabolic	pH 7, 30°C	70% of cypermethrin (50 mg/l) was degraded within 144 h	Ma et al., 2013
*Bacillus* sp. AKD1	Sludge, India	Cypermethrin	Catabolic	pH 8, 37.8°C	86, 73, 67, 51, and 47% of cypermethrin at concentrations of 100, 150, 200, 400, and 500 mg/L, respectively, were degraded in 7 days	Tiwary and Dubey, 2016
*Bacillus* sp. DG-02	Pyrethroid-manufacturing wastewater treatment system, China	Fenpropathrin	Catabolic	pH 7.5, 30°C	100, 93.3, 90.4, 87.6, 84.7, 80.5, 75.8, 67.2, and 61% of bifenthrin at concentrations of 25, 50, 100, 200, 400 600, 800, 1000, and 1200 mg/L, respectively, were degraded in 72 h	Chen et al., 2012a, 2014
		Cypermethrin			89.2% of pesticide (50 mg/L) was removed within 72 h	
		Cyfluthrin			About 86% of pesticide (50 mg/L) was degraded in 72 h	
		λ-Cyhalothrin			Degradation (50 mg/L) reached 82.7% within 72 h	
		Deltamethrin			94.1% of deltamethrin (50 mg/L) was degraded during 72 h	
		Bifenthrin			65.1% of insecticide (50 mg/L) was removed within 72 h	
		Permethrin			63.6% of permethrin (50 mg/L) was degraded in 72 h	
*Bacillus* sp. ISTDS2	Pulp effluent and sludge, India	Cypermethrin	Catabolic	pH 7, 30°C	Almost complete degradation (50 mg/L) within 180 h	Sundaram et al., 2013
*Bacillus* sp. SG2	Contaminated soil, India	Cypermethrin	Catabolic	pH 7, 32°C	Almost 82% of cypermethrin (50 mg/L) was degraded in 15 days	Bhatt et al., 2016
*Bacillus amyloliquefaciens* AP01	Contaminated soil, Korea	Cypermethrin	Catabolic, co-metabolic	pH 7, 30°C	About 45% of cypermethrin (50 mg/L) was removed within 5 days	Lee et al., 2016
*Bacillus cereus (SM-3)*	Soil and sewage sludge, UK	Permethrin	Co-metabolic	pH 7, 30°C	50–90% degradation (20 mg/L) in the presence of Tween 80 within 2 weeks (depended on the type of isomer)	Maloney et al., 1988
*Bacillus cereus Y1*	Deltamethrin-contaminated soil, China	Deltamethrin	Catabolic	pH 7, 30°C	The dissipation rates were 99.4 and 22.8% in 96 h when the initial concentrations were 10 and 100 mg/L, respectively	Zhang et al., 2016
*Bacillus cereus* ZH-3	Activated sludge, China	Cypermethrin	Catabolic	pH 7.5, 28°C	78.4% of cypermethrin (50 mg/l) was degraded within 72 h	Chen et al., 2012b
*Bacillus licheniformis* B-1	Soil in a tea garden, China	Cypermethrin	Catabolic	pH 7–7.5, 30°C	Almost 50% of cypermethrin (100 mg/L) was removed within 72 h	Liu et al., 2014
*Bacillus megaterium* JCm2	Contaminated soil, Pakistan	Cypermethrin	Catabolic	pH 7, 30°C	89% of pesticide (100 mg/L) was degraded in 10 days	Akbar et al., 2015b
		Bifenthrin			About 75% of pesticide (100 mg/L) was degraded in 7 days	
		Cyhalothrin			Approximately 10% removal (100 mg/L) within 7 days	
		Deltamethrin			About 83% of pesticide (100 mg/L) was degraded in 7 days	
*Bacillus subtilis* BSF01	Activated sludge from pesticide-manufacturing wastewater treatment system, China	Cypermethrin	Catabolic	pH 6.7, 34.5°C	93.9, 89.4, and 84.7% degradation at concentrations of 25, 50, and 100 mg/L, respectively, within 7 days	Xiao et al., 2015
		Deltamethrin			86.9 of deltamethrin (50 mg/L) was degraded during 7 days	
		Cyfluthrin			86.5 of initial dose (50 mg/L) was removed within 7 days	
		Cyhalothrin			About 77% of pesticide (50 mg/L) was degraded in 7 days	
		Cypermethrin			89.2% degradation (50 mg/L) within 7 days	
*Bacillus thuringiensis* ZS-19	Activated sludge from pyrethroid-manufacturing wastewater treatment system, China	Cyhalothrin	Catabolic	pH 7.5, 30°C	100, 95.5, 87.4, 84.0, and 82.1% of cyhalothrin at concentrations of < 100, 200, 400, 600, and 800 mg/L, respectively, were degraded in 72 h	Chen et al., 2015
		Fenpropathrin			Nearly 98% (100 mg/L) was degraded in 72 h	
		Deltamethrin			92.4% of initial dose (100 mg/L) was removed within 72 h	
		Cypermethrin			About 81% of pesticide (100 mg/L) was degraded in 72 h	
		Cyfluthrin			86% removal (100 mg/L) after 72 h of incubation	
		Bifenthrin			50.9% degradation (100 mg/L) after 72 h	
*Brevibacillus parabrevis* JCm4	Contaminated soil, Pakistan	Cypermethrin	Catabolic	pH 7, 30°C	28% removal (100 mg/L) within 10 days	Akbar et al., 2015b
*Brevibacillus parabrevis* FCm9	Contaminated soil, Pakistan	Cypermethrin	Catabolic	pH 7, 30°C	Almost 95% degradation (100 mg/L) in 10 days	Akbar et al., 2015a
		Bifenthrin			About 89% of pesticide (100 mg/L) was degraded in 7 days	
		Cyhalothrin			Approximately 60% removal (100 mg/L) within 7 days	
		Deltamethrin			About 82% of pesticide (100 mg/L) was degraded in 7 days	
*Brevibacterium aureum* DG-12	Activated sludge from wastewater treatment system, China	Cyfluthrin	Catabolic	pH 7, 27°C	87.4% degradation (50 mg/L) within 5 days; tolerated cyfluthrin (25–600 mg/L)	Chen et al., 2013
		Cyhalothrin			89.1% degradation (50 mg/L) within 5 days	
		Fenpropathrin			82.6% degradation (50 mg/L) within 5 days	
		Deltamethrin			80.9% degradation (50 mg/L) within 5 days	
		Bifenthrin			80.1% degradation (50 mg/L) within 5 days	
		Cypermethrin			78.3% degradation (50 mg/L) within 5 days	
*Catellibacterium* sp. CC-5	Soil with history of pyrethroid application, China	Cypermethrin	Catabolic	pH 7, 30°C	About 90% of cypermethrin (50, 100, and 200 mg/L) was degraded within 7 days; at 500 and 600 mg/L, 68 and 56% degradation was achieved	Zhao et al., 2013
		Fenvalerate			83% removal (100 mg/L) within 7 days	
		Fenpropathrin			Nearly 81% degradation (100 mg/L) after 7 days	
		Deltamethrin			More than 90% degradation (100 mg/L) in 7 days	
		Permethrin			73% degradation (100 mg/L) within 7 days	
		Cyhalothrin			About 56% of pesticide (100 mg/L) was degraded in 7 days	
*Clostridium* sp. ZP3	Sludge, China	Fenpropathrin	Catabolic, co-metabolic	pH 7.5, 35°C	Slightly degradation (12.6%) of fenpropathrin (100 mg/L) within 12 days	Zhang S. et al., 2011
*Lysinibacillus sphaericus* FLQ-11-1	Activated sludge, China	Cyfluthrin	Catabolic	pH 7, 35°C	Approximately 80% removal (50 mg/L) within 5 days	Hu et al., 2014
*Micrococcus* sp. CPN 1	Pesticide-contaminated soil, India	Cypermethrin	Catabolic	pH 7, 30°C	About 90% removal (1000 mg/L) after 8 days	Tallur et al., 2008
*Ochrobactrum anthropi* JCm1	Contaminated soil, Pakistan	Cypermethrin	Catabolic	pH 7, 30°C	Nearly 91% degradation (100 mg/L) in 10 days	Akbar et al., 2015b
		Bifenthrin			About 70% of pesticide (100 mg/L) was degraded in 7 days	
		Cyhalothrin			Approximately 39% removal (100 mg/L) within 7 days	
		Deltamethrin			About 65% of pesticide (100 mg/L) was degraded in 7 days	
*Ochrobactrum lupini* DG-S-01	Activated sludge from a pyrethroid-manufacturer, China	Cypermethrin	Catabolic, co-metabolic	pH 7, 30°C	Over 90% degradation (50 mg/L) within 5 days	Chen et al., 2011b
		Cyfluthrin			80.8% removal (50 mg/L) within 5 days	
		Fenpropathrin			74.4% removal (50 mg/L) within 5 days	
		Cyhalothrin			56.2% removal (50 mg/L) within 5 days	
		Deltamethrin			43% removal (50 mg/L) within 5 days	
*Ochrobactrum haematophilum* JCm7	Contaminated soil, Pakistan	Cypermethrin	Catabolic	pH 7, 30°C	78% of cypermethrin (100 mg/L) was degraded in 10 days	Akbar et al., 2015b
*Ochrobactrum tritici* pyd-1	Soil contaminated with synthetic pyrethroids from chemical factory, China	Fenpropathrin	Catabolic	pH 7, 30°C	100% degradation (100 mg/L) within 6 days	Wang et al., 2011
		Permethrin			100% degradation (100 mg/L) within 72 h	
		Cypermethrin			100% degradation (100 mg/L) within 6 days	
		Fenvalerate			100% degradation (100 mg/L) within 6 days	
		Cyhalothrin			About 85% degradation (100 mg/L) within 6 days	
		Deltamethrin			About 70% degradation (100 mg/L) within 6 days	
		Bifenthrin			About 50% degradation (100 mg/L) within 6 days	
*Pseudomonas aeruginosa* CH7	Activated sludge, China	Cypermethrin	Catabolic	pH 7, 29.4°C	About 90% of cypermethrin (100 mg/L) was degraded within 12 days	Zhang C. et al., 2011
*Pseudomonas aeruginosa* JCm8	Contaminated soil, Pakistan	Cypermethrin	Catabolic	pH 7, 30°C	46% of cypermethrin (100 mg/L) was degraded in10 days	Akbar et al., 2015b
*Pseudomonas aeruginosa* JQ-41	Pyrethroid-treated soil, China	Fenpropathrin	Co-metabolic	pH 7, 30°C	91.7% degradation (50 mg/L) within 7 days	Song et al., 2015
		Cypermethrin			87.2% degradation (50 mg/L) within 7 days	
		Deltamethrin			90.4% degradation (50 mg/L) within 7 days	
		Bifenthrin			70.1% degradation (50 mg/L) within 7 days	
		Cyhalothrin			74.8% degradation (50 mg/L) within 7 days	
*Pseudomonas fluorescens* (SM-1)	Soil and sewage sludge, UK	Permethrin	Co-metabolic	pH 7, 30°C	20–55% degradation (20 mg/L) in the presence of Tween 80 within 4 weeks (depended on the type of isomer)	Maloney et al., 1988
*Pseudomonas* sp. (*Pseudomonas fluorescens*)	Pyrethroid-contaminated soil, United Kingdom	Cypermethrin	Co-metabolic	pH 7, 25°C	37.2% of cypermethrin (50 mg/L) in the presence of sucrose was degraded within 96 h	Grant et al., 2002; Grant and Betts, 2004
*Pseudomonas stutzeri* S1	Pyrethroid-contaminated soil, India	Cyfluthrin	Catabolic,	pH 7, 28°C	About 94% of cyfluthrin (50 mg/L) was degraded within 8 days (1-day lag phase)	Saikia et al., 2005
*Rhodococcus* sp. JCm5	Contaminated soil, Pakistan	Cypermethrin	Catabolic	pH 7, 30°C	100% of cypermethrin (100 mg/L) was degraded in 10 days	Akbar et al., 2015b
		Bifenthrin			About 93% of pesticide (100 mg/L) was degraded in 7 days	
		Cyhalothrin			Approximately 65% removal (100 mg/L) within 7 days	
		Deltamethrin			About 85% of pesticide (100 mg/L) was degraded in 7 days	
*Serratia* spp. JC1	Activated sludge, China	Cypermethrin	Catabolic	pH 7.6, 31°C	92% degradation (100 mg/L) within 10 days	Zhang et al., 2010
*Serratia* spp. JCN13	Activated sludge, China	Cypermethrin	Catabolic	pH 8, 34°C	Complete degradation (100 mg/L) after 8 days (higher cell surface hydrophobicity in comparison to strain JC1)	Zhang et al., 2010
*Serratia* sp. (*Serratia plymuthica*)	Pyrethroid-contaminated soil, United Kingdom	Cypermethrin	Co-metabolic	pH 7, 25°C	34.2% of cypermethrin (50 mg/L) in the presence of sucrose was degraded within 96 h	Grant et al., 2002; Grant and Betts, 2004
*Serratia marcescens* DeI-1	Deltamethrin-treated soil, Poland	Deltamethrin	Catabolic	pH 7.2, 30°C	Degradation of deltamethrin (50 mg/L) reached 88.3% after 10 days	Cycoń et al., 2014
*Serratia marcescens* DeI-2	Deltamethrin-treated soil, Poland	Deltamethrin	Catabolic	pH 7.2, 30°C	82.8% of deltamethrin (50 mg/L) was degraded within 10 days	Cycoń et al., 2014
*Serratia nematodiphila* CB2	Cypermethrin-treated soil, India	Cypermethrin	Catabolic	pH 7, 30°C	Nearly, 98% of cypermethrin (100 mg/L) was degraded in 7 days	Tyagi and Prashar, 2015
*Sphingobium* sp. JZ-2	Sludge from wastewater treatment system, China	Fenpropathrin	Catabolic	pH 7, 30°C	100% removal (50 mg/L) within 5 days	Guo et al., 2009
		Cypermethrin			About 90% degradation (50 mg/L) within 5 days	
		Permethrin			Nearly 90% degradation (50 mg/L) within 5 days	
		Fenvalerate			About 90% degradation (50 mg/L) within 5 days	
		Deltamethrin			Approximately 90% degradation (50 mg/L) within 5 days	
		Cyhalothrin			Nearly 70% degradation (50 mg/L) within 5 days	
*Sphingobium* sp. JQL4-5	Sludge from wastewater treatment system, China	Bifenthrin	Catabolic	pH 7, 30°C	25% of bifenthrin (50 mg/L) was degraded after 4 day	Yuanfan et al., 2010
		Cypermethrin			36.5% of cypermethrin (100 mg/L) was removed after 48 h	
		Bifenthrin			30.6% of bifenthrin (100 mg/L) was degraded within 48 h	
		Fenvalerate			More than 90% of initial concentration (100 mg/L) was degraded within 48 h	
		Deltamethrin			51.2% degradation (100 mg/L) during 48 h	
		Cyhalothrin			Degradation (100 mg/L) reached 13.3% after 48 h	
*Sphingomonas* sp. JCm3	Deltamethrin-treated soil, Poland	Cypermethrin	Catabolic	pH 7, 30°C	Approximately 34% removal (100 mg/L) within 10 days	Akbar et al., 2015b
*Sphingomonas* sp. RCm6	Deltamethrin-treated soil, Poland	Cypermethrin	Catabolic	pH 7, 30°C	Nearly 92% degradation (100 mg/L) in 10 days	Akbar et al., 2015a
		Bifenthrin			About 83% of pesticide (100 mg/L) was degraded in 7 days	
		Cyhalothrin			Approximately 58% removal (100 mg/L) within 7 days	
		Deltamethrin			About 70% of pesticide (100 mg/L) was degraded in 7 days	
*Stenotrophomonas* sp. ZS-S-01	Activated sludge from pyrethroid- wastewater treatment system, China	Fenvalerate	Catabolic	pH 7, 30°C	Complete degradation (50 mg/L) within 6 days	Chen et al., 2011c
		Deltamethrin			Complete degradation (50 mg/L) within 5 days	
		Cypermethrin			86.7% degradation (50 mg/L) within 5 days	
		Cyfluthrin			Degradation (50 mg/L) reached 85.0% within 5 days	
		Cyhalothrin			60.3% of cyhalothrin (50 mg/L) was removed within 5 days	
*Streptomyces* sp. HU-S-01	Wastewater sludge, China	Cypermethrin	Catabolic	pH 7.5, 26–28°C	About 90% degradation of cypermethrin (50 mg/L) was achieved within 24 h	Lin et al., 2011
*Streptomyces aureus* HP-S-01	Activated sludge from wastewater treatment system, China	Cypermethrin	Catabolic	pH 7.5, 28°C	69.3% of cypermethrin (50 mg/L) was removed within 72 h	Chen et al., 2012c
		Deltamethrin	Catabolic, co-metabolic	pH 7.8, 27°C	99% degradation (50 mg/L) within 4 day	Chen et al., 2011d
		Cyfluthrin	Catabolic	pH 7.8, 27°C	Complete degradation (50 mg/L) during 5 days	
		Bifenthrin			Complete degradation (50 mg/L) within 5 days	
		Fenvalerate			Complete degradation (50 mg/L) during 5 days	
		Fenpropathrin			95% degradation (50 mg/L) within 5 days	
		Permethrin			Degradation (50 mg/L) reached 87.4% after 5 days	

**Table 4:** **Pyrethroid-degrading fungi isolated from contaminated sites and their degradation potential in liquid media**.

**Strain**	**Source**	**Degraded pyrethroid**	**Mode of action**	**Optimal conditions**	**Comments**	**References**
*Aspergillus niger*	Type Culture Lab, IARI, India	Cyfluthrin	Co-metabolic	pH 6.5, 28°C	10% of initial dosage (5 mg/L) was degraded after 30 days	Saikia and Gopal, 2004
*Aspergillus terricola*	Type Culture Lab, IARI, India	Cyfluthrin	Co-metabolic	pH 6.5, 28°C	About 25% degradation (5 mg/L) after 30 days	Saikia and Gopal, 2004
*Candida pelliculosa* ZS-02	Activated sludge from wastewater treatment system, China	Bifenthrin	Catabolic	pH 7.2, 32°C	Complete degradation of bifenthrin at concentration of 100 mg/L within 5 days; At concentrations of 200, 300, and 400 mg/L, the degradation rates reached 97.1, 95.8, and 91.3% after 5 days, respectively	Chen et al., 2012d
		Cyfluthrin			More than 94.8% degradation (50 mg/L) after 5 days	
		Deltamethrin			93.4% degradation (50 mg/L) within 5 days	
		Fenvalerate			93% of fenvalerate (50 mg/L) was degraded after 5 days	
		Cypermethrin			87.7% degradation (50 mg/L) within 5 days	
		Fenpropathrin			Nearly 51% degradation (50 mg/L) after 7 days	
*Cladosporium* sp. HU	Activated sludge from wastewater treatment system, China	Fenvalerate	Catabolic, co-metabolic	pH 7.2, 26°C	Complete degradation of fenvalerate at 50–400 mg/L within 5 days; degradation accelerated in the presence of sucrose; ability to degrade main metabolite, i.e., 3-phenoxybenzaldehyde	Chen et al., 2011a
		Fenpropathrin	Catabolic	pH 7.2, 26°C	Complete degradation (100 mg/L) after 5 days	
		Cypermethrin			Complete degradation (100 mg/L) after 5 days	
		Deltamethrin			94.6% degradation (100 mg/L) after 5 days	
		Bifenthrin			92.1% degradation (100 mg/L) after 5 days	
		Permethrin			91.6% degradation (100 mg/L) after 5 days	
*Phanerocaete chrysosporium*	Type Culture Lab, IARI, India	Cyfluthrin	Co-metabolic	pH 6.5, 28°C	About 60% degradation (5 mg/L) after 30 days	Saikia and Gopal, 2004
*Trichoderma viridae* (5-2)	Type Culture Lab, IARI, India	Cyfluthrin	Co-metabolic	pH 6.5, 28°C	80–83% degradation (5 mg/L) was observed in 10 days (maximum degradation within 7 days)	Saikia and Gopal, 2004
*Trichoderma viridae* (2211)	Type Culture Lab, IARI, India	Cyfluthrin	Co-metabolic	pH 6.5, 28°C	About 60% degradation (5 mg/L) after 30 days	Saikia and Gopal, 2004

Among the strains tested, bacteria that belong to the genera *Bacillus, Brevibacillus, Ochrobactrum, Pseudomonas, Serratia*, and *Sphingobium* were found to be very metabolically active microorganisms that are capable of degrading various pyrethroids (Table 3). For example, *Bacillus* sp. AKD1 (Tiwary and Dubey, 2016) and *Bacillus* sp. DG-02 (Chen et al., 2012a) nearly completely degraded cypermethrin and bifenthrin, respectively, applied at the concentration of 100 mg/L during a few days. The DG-02 strain was also able to degrade 61% of extremely high concentration of bifenthrin (1200 mg/L) within seven days (Chen et al., 2012a). Similarly, *Ochrobactrum anthropi* strain JCm1 (Akbar et al., 2015b), *Ochrobactrum lupini* strain DG-S-01 (Chen et al., 2011b), *Ochrobactrum tritici* strain pyd-1 (Wang et al., 2011), and *Serratia nematodiphila* strain CB2 (Tyagi and Prashar (2015) were found to be capable of directly utilizing more than 90% of the initial dose of cypermethrin (100 mg/L) within 5–10 days. Also fungi have been found as the effective pyrethroids degraders (Table 4). For example, *Cladosporium* sp. strain HU was able to completely remove fenvalerate (50–400 mg/L) from the medium within 5 days (Chen et al., 2011a). In another study, Chen et al. (2012d) showed a high efficiency of *Candida pelliculosa* ZS-02 to degrade a wide range of concentrations of bifenthrin (100–400 mg/L) in a liquid medium within the same tame.

For the efficient bioremediation of pyrethroid-contaminated soils, it would be advantageous if microorganisms could be used against many pyrethroids. Since the structure of pyrethroids is generally similar, it is expected that these microorganisms would be capable of degrading various pyrethroids (Cycoń et al., 2014). It has been reported that some bacteria (Table 3) and fungi (Table 4) have been found to be capable of directly degrading not only one but a wide spectrum of pyrethroids as the sole source of carbon. Furthermore, some pyrethroid-degrading microorganisms were also able to utilize the metabolites that arise from the degradation of parental compounds (Tables 3, 4). For example, the fenvalerate-degrading *Stenotrophomonas* sp. strain ZS-S-01 (Chen et al., 2011c) and the deltamethrin-degrading *Streptomyces aureus* strain HP-S-01 (Chen et al., 2011d) were capable of degrading pyrethroids such as fenpropathrin, bifenthrin, β-cypermethrin, cyhalothrin, or permethrin with a wide range of efficacies. In most cases, both strains completely degraded pyrethroids (50 mg/L) within 5 days of incubation. Moreover, strains ZS-S-01 and HP-S-01 also eliminated 3-phenoxybenzoic acid and 3-phenoxybenzaldehyde from liquid media, respectively (Chen et al., 2011c,d). In another study, Guo et al. (2009) demonstrated that *Sphingobium* sp. strain JZ-2 was capable of degrading up to six different pyrethroids used at a concentration of 50 mg/L. Biodegradation results revealed that strain JZ-2 utilized 100% of fenpropathrin, nearly 90% of cypermethrin, permethrin, fenvalerate, and deltamethrin and about 70% of cyhalothrin within 5 days of incubation. *O. tritici* strain pyd-1 was also found to be capable of utilizing seven pyrethroids with different efficiencies (Wang et al., 2011). Among the pyrethroids tested, the fastest degradation occurred in the case of permethrin; 100% of the initial concentration (100 mg/L) was removed by strain pyd-1 after 72 h. The ability to degrade more than one pyrethroid compound was also reported for *Bacillus* sp. DG-02 (Chen et al., 2014), *Bacillus subtilis* BSF01 (Xiao et al., 2015), and *Brevibacillus parabrevis* FCm9 (Akbar et al., 2015a). Similarly, the fungi were found to be capable of degrading a wide spectrum of pyrethroids. For example, *C. pelliculosa* strain ZS-02 utilized bifenthrin, cyfluthrin, deltamethrin, fenvalerate, or cypermethrin with high efficiency (87.7–95.8%) within 5 days (Chen et al., 2012d).

Some studies have also revealed that the use of a mixed culture of bacterial strains resulted in an enhancement of the degradation of pyrethroids. For example, Chen et al. (2012b) used two pyrethroid-degrading strains, *Bacillus cereus* ZH-3 and *S. aureus* HP-S-01, in a mixed culture for the degradation of cypermethrin. As was revealed by the authors, although strains ZH-3 and HP-S-01 together exhibited some degradation of cypermethrin, the efficiency of the cypermethrin removal decreased over time. A biodegradation experiment showed that a mixed culture metabolized 73.1% of the cypermethrin concentration (50 mg/L) within 24 h, while in the same period, individual strains ZH-3 and HP-S-01 alone degraded only 37.5 and 23.0% of the added cypermethrin, respectively. The efficiency of the cypermethrin removal of a mixed culture significantly increased with incubation time, reaching 95.7 and 100% within 48 and 72 h, respectively. However, the individual strains ZH-3 and HPS-01 alone could not degrade the added cypermethrin and only 78.4 and 69.3% of the initial dose, respectively, was degraded at the end of the experiment (Chen et al., 2012b). Similarly, Liu et al. (2014) demonstrated that the cooperation of *Bacillus licheniformis* B-1 and *Sphingomonas* sp. SC-1 is capable of directly degrading cypermethrin with a higher efficiency. The half-life of cypermethrin using two strains was shortened from 71.9 to 35.7 h compared to using only strain B-1.

Some studies have shown that not all pyrethroid-degrading microorganisms have a high efficiency in degrading certain pyrethroids. For example, Akbar et al. (2015b) demonstrated that more than 75% of the initial dose (100 mg/L) of cypermethrin, bifenthrin, or deltamethrin was degraded by *Bacillus megaterium* JCm2 in 7 days, whereas in the same period, only 10% of cyhalothrin was removed from the medium. Similarly, Yuanfan et al. (2010) showed that *Sphingobium* sp. JQL4-5 was characterized by a lower degradation activity against some pyrethroids. It could only utilize 51.2% of deltamethrin, 36.5% of cypermethrin, 25% of bifenthrin and 13.3% of cyhalothrin, respectively. Also, Saikia and Gopal (2004) demonstrated that fungal strains *Aspergillus niger* and *Aspergillus terricola* degraded only 10 and 25% of the initial dose of cyfluthrin (5 mg/L), respectively, within 30 days. A slower degradation rate as compared to bifenthrin, cyfluthrin, deltamethrin, fenvalerate, or cypermethrin was achieved in the case of fenpropathrin for *C. pelliculosa* strain ZS-02 as well (Chen et al., 2012d).

### Bioremediation of pyrethroid-contaminated soil

The microorganisms showing a potential of pyrethroids degradation in liquid media can also degrade them in soil. However, the potential of these microorganisms to utilize pyrethroids in soils and their use in the remediation of pyrethroid-contaminated soils has only been confirmed for some bacteria of the genera *Acinetobacter* (Akbar et al., 2015a), *Bacillus* (Sundaram et al., 2013; Chen et al., 2014; Zhang et al., 2016), *Brevibacillus* (Akbar et al., 2015a), *Catellibacterium* (Zhao et al., 2013), *Ochrobactrum* (Akbar et al., 2015b), *Rhodococcus* (Akbar et al., 2015b), *Serratia* (Cycoń et al., 2014), *Sphingomonas* (Akbar et al., 2015a), *Stenotrophomonas* (Chen et al., 2011c), *Streptomyces* (Chen et al., 2012c), and fungi including *Candida* (Chen et al., 2012d; Table 5).

**Table 5:** **The degradation potential of pyrethroid-degrading microorganisms in soils**.

**Microorganism**	**Degraded pesticide**	**Dosage (mg/kg)**	**Type of soil**	**Type of experiment**	**Inoculum size (cfu/g)**	**Comments**	**References**
*Acinetobacter calcoaceticus* MCm5	Cypermethrin	200	No data	In laboratory, 30°C	1 × 10^7^	Almost 90% of cypermethrin was removed from inoculated soil within 42 days (*t*_1/2_ 17.1 days) whereas in control soil (non-sterilized) 43.7% of initial dose was degraded (*t*_1/2_ 48.5 days)	Akbar et al., 2015a
*Bacillus* sp. DG-02	Fenpropathrin	50	Sandy loam	In laboratory, 30°C	1 × 10^8^	The value of *t*_1/2_ for fenpropathrin in inoculated soil was shortened, giving value of 5.4 days as compared to the control (37.1 days)	Chen et al., 2014
*Bacillus* sp. ISTDS2	Cypermethrin	100	Loam	In laboratory, 30°C	5 × 10^7^	Complete degradation of cypermethrin in inoculated sterile soil within 30 days	Sundaram et al., 2013
*Bacillus cereus* Y1	Deltamethrin	10	Soil from vegetable farmland	In laboratory, 30°C	1 × 10^10^	The dissipation rate of deltamethrin in inoculated soil was 74.9% during 25 days (*t*_1/2_ 5.2 days)–in control 45.1% (*t*_1/2_ 8.3 days)	Zhang et al., 2016
*Bacillus licheniformis* B-1	Cypermethrin	20	Soil from vegetable farmland	In laboratory, 22°C	1 × 10^8^	The degradation of cypermethrin was 54% (*t*_1/2_ 19.9 days) in inoculated soil within 25 days (in control only 14%, *t*_1/2_ 5.2 days)	Liu et al., 2014
*Bacillus megaterium* JCm2	Cypermethrin	200	No data	In laboratory, 30°C	1.6 × 10^7^	Nearly 88% of cypermethrin was removed from inoculated soil within 42 days (*t*_1/2_ 16.3 days)—in non-sterilized control soil (*t*_1/2_ 48.5 days)	Akbar et al., 2015b
*Brevibacillus parabrevis* FCm9	Cypermethrin	200	No data	In laboratory, 30°C	1 × 10^7^	Almost 90% of cypermethrin was removed from inoculated soil within 42 days (*t*_1/2_ 9.6 days) whereas in control soil (non-sterilized) 43.7% of initial dose was degraded (*t*_1/2_ 48.5 days)	Akbar et al., 2015a
*Candida pelliculosa* ZS-02	Bifenthrin	50	Sandy loam	In laboratory, 32°C	1 × 10^7^	Almost 75% of bifenthrin was removed from inoculated soil within 10 days (*t*_1/2_ 4.9 days) whereas in control soil (non-sterilized) 8.4% of initial dose was degraded (*t*_1/2_ 78.8 days)	Chen et al., 2012d
*Catellibacterium* sp. CC-5	Cypermethrin	50	Soil from grass-covered field	In laboratory, 30°C	5 × 10^6^	Almost 86% of cypermethrin was removed from inoculated soil within 10 days (*t*_1/2_ 3.4 days) whereas in control soil (non-sterilized) 26.9% of initial dose was degraded (*t*_1/2_ 21.6 days)	Zhao et al., 2013
*Ochrobactrum anthropi* JCm1	Cypermethrin	200	No data	In laboratory, 30°C	1.6 × 10^7^	93.4% of cypermethrin was removed from inoculated soil within 42 days (*t*_1/2_ 13.4 days)–in non-sterilized control soil (*t*_1/2_ 48.5 days)	Akbar et al., 2015b
*Rhodococcus* sp. JCm5	Cypermethrin	200	No data	In laboratory, 30°C	1.6 × 10^7^	100% of cypermethrin was removed from inoculated soil within 42 days (*t*_1/2_ 8.6 days)–in non-sterilized control soil (*t*_1/2_ 48.5 days)	Akbar et al., 2015b
*Serratia marcescens* DeI-1	Deltamethrin	100	Sand, Sandy loam, Silty loam, Silt	In laboratory, 30°C	3 × 10^6^	61–82% of the initial dose of deltamethrin was removed in inoculated soils after 84 days (DT50 27.0–47.1 days)–in non-sterile control soils (41.8-59.8%–DT50 68.8–105.3 days)	Cycoń et al., 2014
*Serratia marcescens* DeI-2	Deltamethrin	100	Sand, Sandy loam, Silty loam, Silt	In laboratory, 30°C	3 × 10^6^	70–92% of the initial dose of deltamethrin was removed in inoculated soils after 84 days (DT50 32.8–59.3 days)–in non-sterile control soils (41.8-59.8%–DT50 68.8–105.3 days)	Cycoń et al., 2014
*Sphingomonas* sp. RCm6	Cypermethrin	200	No data	In laboratory, 30°C	1 × 10^7^	Almost 100% of cypermethrin was removed from inoculated soil within 42 days (*t*_1/2_ 14.4 days) whereas in control soil (non-sterilized) 43.7% of initial dose was degraded	Akbar et al., 2015a
*Stenotrophomonas* sp. ZS-S-01	Fenvalerate	50	Silty loam	In laboratory, 30°C	1 × 10^7^	93.4% of fenvalerate was degraded within 9 days (*t*_1/2_ 2.3 days)–in control only 28.7% (*t*_1/2_ 19.2 days); ability to degrade the main metabolite, i.e., 3-phenoxybenzoic acid	Chen et al., 2011c
*Streptomyces aureus* HP-S-01	Cypermethrin	50	Sandy loam	*In situ*, 24–30°C	1 × 10^6^	81.1% of cypermethrin was removed in bioaugmented soil (*t*_1/2_ 4.1 days)–in control 32.1% (*t*_1/2_ 17.2 days), HP-S-01 strain was also capable of degrading the main metabolite, i.e., 3-phenoxybenzaldehyde	Chen et al., 2012c

Among the biological approaches, which include attenuation, biostimulation, and bioaugmentation, the last one seems to be the most promising for the removal of pyrethroids and their residues from soil (Chen et al., 2012c; Zhao et al., 2013; Cycoń et al., 2014; Akbar et al., 2015a). The usefulness of bioaugmentation with microorganisms in the clean-up of polluted soil has also been demonstrated some years ago in relation to other pesticides including organochlorinated pesticides (Kataoka et al., 2011; Sáez et al., 2014), organophosphorus pesticides (Cycoń et al., 2013; Aceves-Diez et al., 2015), triazines (Wang et al., 2013; Silva et al., 2015), carbamate (Pimmata et al., 2013), chloroacetamide (Zheng et al., 2012), benzimidazole (Wang et al., 2010), and derivatives of phenoxyacetic acid (Önneby et al., 2014). The promising results of these studies caused an increasing interest in screening new pyrethroid-degrading strains and searching for more effective bioremediation approaches (Chen et al., 2011a; Zhao et al., 2013; Cycoń et al., 2014; Liu et al., 2014; Akbar et al., 2015b; Zhang et al., 2016).

As has been shown in studies on the bioremediation of pyrethroid-contaminated soils, the inoculated bacterial or fungal strains were capable of degrading various pyrethroids with a high efficiency. However, most of these studies were related to the degradation of cypermethrin and were performed under controlled laboratory conditions (Table 5). The dosages used in these studies covering a worst case scenario of pesticide concentrations in soils and showed a very high potential of pyrethroid-degrading microbial strains to eliminate pesticides from polluted soils. The study of Akbar et al. (2015a) showed that cypermethrin at a concentration of 200 mg/kg soil was almost totally removed (90–100%) from soil inoculated (1 × 10^7^ cells/g soil) with *Acinetobacter calcoaceticus* MCm5, *Brevibacillus parabrevis* FCm9 or *Sphingomonas* sp. RCm6 within 42 days. In the same time, the initial concentration of cypermethrin in the non-sterilized control soil was reduced by about 44%. The same authors also showed the ability of *Bacillus megaterium* JCm2, *O. anthropi* JCm1 or *Rhodococcus* sp. JCm5 to degrade cypermethrin in soil. In these cases, the degradation rate of pyrethroids in the bioaugmented soil reached 88–100% during the 42-day experiment (Akbar et al., 2015b). In the field-scale experiment, *S. aureus* HP-S-01 (1 × 10^7^ cells/g soil) inoculated to soil contaminated with β-cypermethrin and its metabolite 3-PBA (both 50 mg/kg soil) quickly adapted to the environment and rapidly removed both compounds from the soil without any lag phases (Chen et al., 2012c). After 10 days, the initial β-cypermethrin and 3-PBA concentrations in the inoculated soils was reduced by 81.1 and 73.5%, respectively, while in the control soils, it decreased by about 32 and 4%, respectively (Chen et al., 2012c).

The positive effect of the bioaugmentation using two isolates of *Serratia marcescens* (DeI-1 and DEI-2) on the removal of pyrethroids from soil was observed by Cycoń et al. (2014). They observed that the degradation of deltamethrin (100 mg/kg soil) in the inoculated soil increased substantially within 84 days of the experiment reaching a final rate of biodegradation at the level of 61–82 and 70–92%, respectively, for soils inoculated with DeI-1 and DEI-2. In comparison, 41.8–59.8% of the initial dose of deltamethrin was removed in the non-inoculated soils. The enhanced degradation of deltamethrin in soil inoculated with *B. cereus* Y1 was also reported by Zhang et al. (2016).

The bioaugmentation with *Stenotrophomonas* sp. ZS-S-01 appeared to be an efficient method for removal of fenvalerate and its hydrolysis product 3-phenoxybenzoic acid both applied at 50 mg/kg soil (Chen et al., 2011c). After 9 days, 93.4% of the initial fenvalerate dose was degraded in the soil inoculated with ZS-S-01 (1 × 10^7^ cells/g soil), while the fenvalerate content decreased by only 28.7% in the control soil with autochthonous microorganisms. Similarly, the initial concentration of 3-phenoxybenzoic acid in the inoculated soil decreased by 81.4% within 10 days, whereas only 9.7% of the applied dose was degraded in the control soil (Chen et al., 2011c).

Similarly, the bioaugmentation of fenpropathrin-contaminated soil with the *Bacillus* sp. DG-02 strain significantly enhanced the disappearance rate of fenpropathrin used at a concentration of 50 mg/kg soil. The half-life value for fenpropathrin in the inoculated soil was shortened to 5.4 days compared to 37.1 days for the control soil without an inoculation (Chen et al., 2014). Another fenpropathrin-degrading bacterium, *Sphingobium* sp. JQL4-5, was used by Yuanfan et al. (2010) to construct a new genetically modified microorganisms (GMM) JQL4-5-*mpd* by introducing the *mpd* genes encoding a methyl parathion hydrolase into its chromosome. This resulted in a multifunctional bacterium that was able to degrade pyrethroid insecticides and methyl parathion indicating the promising potential of the newly constructed strains of bacteria in the bioremediation of pyrethroid-contaminated soils.

Apart from the application of GMM, the simultaneously use of more than one bacterial strain offers a new approach for enhancement removal of pyrethroids from the environment. For example, Liu et al. (2014) found that in soil inoculated with *B. licheniformis* B-1 and *Sphingomonas* sp. SC-1 the degradation rates of cypermethrin and 3-phenoxybenzoic acid was significantly higher than in the control and soil bioaugmented with only strain B-1.

Most studies have primarily been focused on bacterial strains but one study have shown that pyrethroids in soil can be degraded by fungi. This ability is mainly attributed to their lignin-degrading enzymes and thus fungi may be extremely effective in the decomposition of many toxic contaminants (Rhodes, 2013). In a bioaugmentation experiment carried out by Chen et al. (2012d) the fungal strain *C. pelliculosa* ZS-02 (1 × 10^7^ spores/g soil) isolated from activated sludge was capable of degrading bifenthrin at a concentration of 50 mg/kg soil. Almost 75% of the bifenthrin was removed from the inoculated soil within ten days of incubation whereas only 8.4% of initial concentration was degraded in non-sterilized control soil without inoculum within the same period (Chen et al., 2012d).

### Biodegradation pathways of pyrethroids

The primary way that pyrethroids are degraded by microorganisms is *via* ester-bond hydrolysis by carboxylesterases (carboxylic-ester hydrolase, EC 3.1.1.1), which yields carboxylate and alcohol (Sogorb and Vilanova, 2002; Aranda et al., 2014). Carboxylesterases are a family of enzymes that mediate the hydrolysis of a large number of ester-containing pesticides such as carbamates, organophosphates, and pyrethroids (Sogorb and Vilanova, 2002). To date, a few pyrethroid-degrading enzymes have been isolated, purified and characterized—carboxylase from *B. cereus* SM3 (Maloney et al., 1993), pyrethroid hydrolase from *A. niger* ZD11 (Liang et al., 2005), EstP from *Klebsiella* sp. ZD112 (Wu et al., 2006) and PytH (31 kDa) from *Sphingobium* sp. JZ-1 (Guo et al., 2009; Wang et al., 2009). In addition, Pye3 was screened from the metagenome of soil (Li et al., 2008), PytZ and PytY from the genomic library of *O. anthropi* YZ-1 (Zhai et al., 2012; Ruan et al., 2013) and thermostable esterase Sys410 from the Turban Basin metagenomics library (Fan et al., 2012).

The studied pyrethroid hydrolases differed in their chemical structure, molecular mass, optimal pH, and temperature. They are also monomeric with the exception of the tetrameric permethrinase that was isolated from *B. cereus* SM3. Lower molecular weights were reported for PytZ (24.2 kDa) and PytH (31 kDa), moderate ones for the hydrolase from *Aspergillus niger* ZD11 (56 kDa) and permethrinase (61 kDa) from *B. cereus* SM3 and the highest for EstP (73 kDa). Although they displayed high activity and stability over a broad range of temperature and pH, the values of the optimal parameters were slightly different. For example, the optimal temperature and pH for pytZ and pytY were 35°C and 7.5, for pytH 40°C and 7.5 and for the carboxylase from *A. niger* ZD11 45°C. The amino acid sequences of these enzymes have moderate (30–59% identity) with known esterase sequences, with the exception of PytZ and PytY that have 85% similarity (Liang et al., 2005; Zhai et al., 2012; Ruan et al., 2013).

The described pyrethroid-hydrolyzing esterases are broad-spectrum enzymes that can degrade various pyrethroids, which may be related to the similar molecular structure of pyrethroid pesticides. However, they differ in the rate of pesticide hydrolysis. For example, PytH hydrolyzed fenpropathrin, permethrin and cypermethrin at a similar rate but had a significantly lower efficiency for deltamethrin and bifenthrin. Some of these carboxylases not only degraded pesticides but also hydrolyzed the *p*-nitrophenyl esters of medium-short chain fatty acids and the organophosphorus insecticide malathion (Wu et al., 2006). Apart from the pyrethroid carboxylase other enzymes contribute in the degradation of intermediates formed from the parental compounds. For example, in the cell free extract of *O. tritici* strain pyd-1 the activity of 3-phenoxybenzoic acid dehydrogenase, 3-PBA hydroxylase, 4-hydroxy-PBA dioxygenase, protocatechuate-3,4-dioxygenase and *p*-hydroquinone hydroxylase was observed (Wang et al., 2011). All of these enzymes were synthesized by bacterial cell grown in mineral medium supplemented with fenpropathrin, while only pyrethroid hydrolase and 3-phenoxybenzoic acid dehydrogenase activities were also detected in bacteria grown in medium with glucose. This indicates that the two last enzymes are expressed constitutively in strain pyd-1. The activity of other enzymes such as phenol hydroxylase, catechol-1,2-dioxygenase and catechol-2,3-dioxygenase was observed in cell free extract of *Micrococcus* sp. strain CPN 1 during the degradation of cypermethrin (Tallur et al., 2008).

To date, only a few pyrethroid-degrading genes such as *estP* (*Klebsiella* sp. ZD112), *pytH* (*Sphingobium* sp. JZ-1), *pye3* (from the metagenome of soil), *pytZ* and *pytY* (from genomic library of *O*. *anthropi* YZ-1) have been characterized (Wu et al., 2006; Li et al., 2008; Wang et al., 2011; Zhai et al., 2012; Ruan et al., 2013). These genes had very low sequence similarity. The low similarity was even observed in the case of *pytZ* and *pytY* that originated from the same *O. anthropi* YZ-1 strain (Ruan et al., 2013).

The reported ways that pyrethroids are degraded differed in the number of intermediates and proposed degradation pathways are presented in Table 6. The differences probably resulted from the biochemical properties of the microbial strain that was studied, different incubation periods (length of experiment) and the stereoisomers of the pyrethroids. Since the intermediates differ in their stability, the frequency of sample collection for analysis may decide on the compounds detected. The degradation pathways of pyrethroids by the bacteria that are capable of pesticide hydrolysis in some cases are similar but significant differences have also been revealed. To date, the pathways of the microbial degradation of cyfluthrin, fenpropathrin and cypermethrin have mostly been studied in detail (Table 6). It has been reported that the major intermediate metabolites after the hydrolysis of most pyrethroids are 3-phenoxybenzaldehyde (3-PBA) or 3-phenozybenzoic acid (Chen et al., 2011a, 2013; Sundaram et al., 2013). However, some microorganisms that are capable of degrading both parental pyrethroids and 3-PBA were found. Such a capability was reported for *Bacillus* sp. DG-02 (Chen et al., 2014), *S. aureus* HP-S-01 (Chen et al., 2012c) and *Streptomyces* sp. HU-S-01 (Lin et al., 2011). Not all bacterial strains degrade pyrethroids *via* 3-PBA (Table 6). For example, this compound was not detected during cyfluthrin degradation by *Brevibacterium aureum* DG-12 (Chen et al., 2014), *Trichoderma viridea* (5-2) (Saikia and Gopal, 2004) or *Pseudomonas stutzeri* S1 (Saikia et al., 2005). The first two microorganisms hydrolyzed cypermethrin to α-cyano-4-fluorobenzyl-3-(2,2-dichlorovinyl)-2,2-dimethyl cyclopropane carboxylate and α-cyano-4-fluoro-3-phenoxy benzyl alcohol (Table 6). 3-PBA was also not found among intermediate metabolites during the degradation of allethrin by *Acidomonas* sp. (Paingankar et al., 2005; Table 6).

**Table 6:** **Detected metabolites and proposed pathways for degradation of pyrethroids performed by selected microorganisms**.

**Pyrethroid**	**Microorganism**	**Detected metabolites**	**Proposed degradation pathway**	**References**
Allethrin (A)	*Acidomonas* sp.	(B) Allethrolone [2-cyclopenten-1-one, 4-Hydroxy-3-methyl-2(-2-propenyl)] (C) Chrysanthemic acid Lub (D) 2-Ethyl-1,3-dimethyl cyclopent-2-ene-carboxylic acid (E) Cyclopropanecarboxylic acid, 2,2-dimethyl-3-(2-methyl-1-propenyl) (F) 3-Oxobicyclo [4.1.0] heptan-2-one, 4,4,7,7-tetramethyl (G) 2-Propanal (1,1-dimethyl ethyl)-methyl	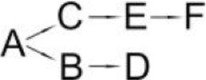	Paingankar et al., 2005
Bifenthrin (A)	*Candida pelliculosa*	(B) Cyclopropanecarboxylic acid (C) 2-Methyl-3-biphenylyl methanol (D) 4-Trifluoromethoxy phenol (E) 2-Chloro-6-fluoro benzylalcohol (F) 3,5-Dimethoxy phenol	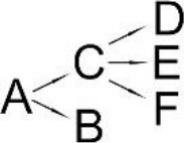	Chen et al., 2012d
Cyfluthrin (A)	*Brevibacterium aureum* DG-12	(B) 2,2,3,3-Tetramethyl-cyclopropanemethanol (C) 4-Fluoro-3-phenoxy-benzoic acid (D) 3,5-Dimethoxy phenol (E) Phenol	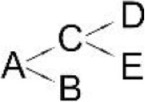	Chen et al., 2013
	*Lysinibacillus sphaericus* FLQ-11-1	(B) 4-fluoro-3-phenoxy-benzoic acid methyl ester (C) Methyl-3-(2,2-dichlorovinyl)-2,2-dimethyl-(1-cyclopropane)-carboxylate (D) Methyl-3-phenoxybenzoate (E) 3-Phenoxy-benzaldehyde (F) Terephthalic (G) Phenol	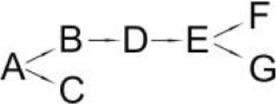	Hu et al., 2014
β-Cyfluthrin (A)	*Pseudomonas stutzeri* S1	(B) α-cyano-4-fluorobenzyl-3-(2,2-dichlorovinyl)-2,2-dimethyl cyclopropane carboxylate (C) α-cyano-4-fluoro-3-phenoxy benzyl alcohol (D) 3(2,2-dichlorovinyl)- 2,2-dimethyl cyclopropane carboxylic acid	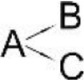	Saikia et al., 2005
	*Trichoderma viridae* (5-2)	(B) α-cyano-4-fluorobenzyl-3-(2,2-dichlorovinyl)-2,2-dimethyl cyclopropane carboxylate/α-cyano-4-fluoro-3-phenoxy benzyl alcohol (C) 3(2,2-dichlorovinyl)- 2,2-dimethyl cyclopropanoic acid	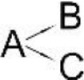	Saikia and Gopal, 2004
Cyhalothrin (A)	*Bacillus thuringiensis* ZS-19	(B) α-Hydroxy-3-phenoxy-benzenacetonitrile (C) (1R, 3R)-trans-2,2-Dimethyl-3-(2-methyl-1-propenyl) cyclopropane-1-carboxylic acid (D) 3-Phenoxy-benzamide (E) 3-Phenoxybenzoic acid (F) 3-Phenozybenzoate (G) Phenol	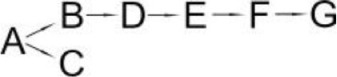	Chen et al., 2015
Cypermethrin (A)	*Bacillus* sp. ISTDS2	(B) (1)α-Hydroxy-3-phenoxy-benzenacetonitrile (C) 3-(2,2-dichloroethenyl)-2,2-dimethyl cyclopropanecarboxylate (D) 3-Phenoxybenzoic acid	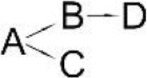	Sundaram et al., 2013
	*Bacillus* sp. SG2	(B) α-Hydroxy-3-phenoxy-benzenacetonitrile (C) 3-(2,2-dichloroethenyl)-2,2-dimethyl cyclopropanecarboxylate (D) 3-Phenoxybenzenaldehyde (E) Hydroxybenzoate (F) 4-Propoyl Benzaldehyde (G) Phenoxy benzoic acid (H) Phenol, M-tert-Butyl (I) Phenol	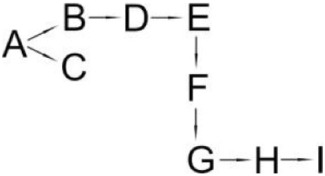	Bhatt et al., 2016
	*Bacillus cereus* ZH-3 + *Streptomyces aureus* HP-S-01	(B) 4-Phenoxyphenol-2,2-dimethyl-propiophenone (C) 3-Phenoxybenzenaldehyde (D) α-Hydroxy-3-phenoxy-benzenacetonitrile	No data	Chen et al., 2012b
	*Bacillus licheniformis* B-1 + *Sphingomonas* sp. SC-1	(B) Cyano -3-phenoxybenzyl alcohol (C) 3-Phenoxybenzaldehyde (D) 3-Phenoxybenzoic acid (E) 2-Phenoxyphenol (F) Catechol (G) Muconic acid	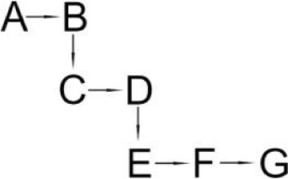	Liu et al., 2014
	*Catellibacterium* sp. CC-5	(B) α-Hydroxy-3-phenoxy-benzenacetonitrile (C) 3-Phenoxybenzaldehyde	No data	Zhao et al., 2013
	*Micrococcus* sp. CPN 1	(B) Cyano -3-phenoxybenzylalcohol (C) 3-(2,2-dichlorovinyl)-2,2-dimethylcyclopropanecarboxylate (D) Cyano-Phenoxybenzaldehyde (E) 3-Phenoxybenzoic acid (F) Phenol (G) Protocatechuic (H) Catechol (I) *cis*,*cis*-Muconic acid (J) 3-Carboxy-cis, cis muconic acid	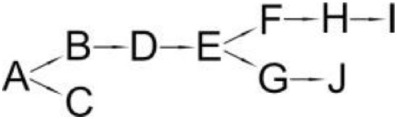	Tallur et al., 2008
β-Cypermethrin (A)	*Bacillus subtilis* BSF01	(B) α-Hydroxy-3-phenoxy-benzenacetonitrile l (C) 3-(2,2-dichloroethenyl)-2,2-dimethylcyclopropanecarboxylate (D) 3-Phenoxybenzaldehyde (E) 3-Phenoxybenzoic acid (F) 3,5-Dimetoxyphenol	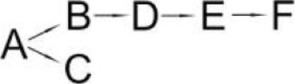	Xiao et al., 2015
	*Streptomyces aureus* HP-S-01	(B) 3-Phenoxybenzenaldehyde (C) α-Hydroxy-3-phenoxy-benzenacetonitrile	No data	Chen et al., 2012c
Deltamethrin (A)	*Streptomyces aureus* HP-S-01	(B) α-Hydroxy-3-phenoxy-benzenacetonitrile (C) 3-Phenoxybenzaldehyde (D) 2-Hydroxy-4-methoxy benzophenone	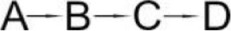	Chen et al., 2011d
Fenpropathrin (A)	*Clostridium* sp. ZP3	(B) Benzyl alcohol (C) Benzene-methanol (D) 3,5-dimethylamphetamine	No data	Zhang S. et al., 2011
	*Bacillus cereus* ZH-3	(B) α-Hydroxy-3-phenoxy-benzenacetonitrile (C) 3-Phenoxybenzenaldehyde (D) Phenol	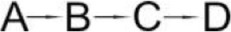	Liu et al., 2015
	*Bacillus* sp. DG-02	(B) 2,2,3,3-Tetramethyl cyclopropanecarboxylic acid phenyl ester (C) 3,4-Dihydroxy-benzoic acid (D) 3-Phenoxybenzoate (E) 3,4-Dimethoxy phenol (F) 3-Phenoxybenzaldehyde (G) α-Hydroxy-3-phenoxy-benzenacetonitrile (H) Phenol	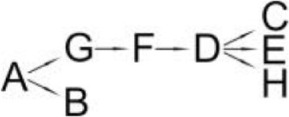	Chen et al., 2014
	*Ochrobactrum tritici* pyd-1	(B) cyano-3-phenoxybenzylalcohol (C) 2,2,3,3-Tetramethyl cyclopropanecarboxylic acid (D) 3-Phenoxybenzaldehyde (E) 3-Phenoxybenzoic acid (F) 4-Hydroxy-3-phenoxybenzoic acid (G) Protocatechuate (H) p-Hydroquinone (I) 1,2,4-Benzenetriol (J) Maleylacetate (K) 3-Oxoadipate	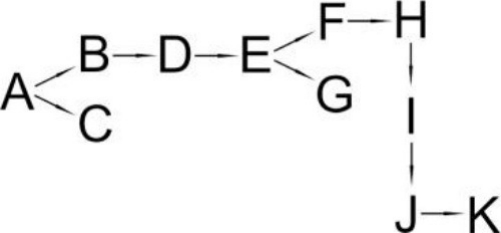	Wang et al., 2011
	*Pseudomonas aeruginosa* JQ-41	(B) α-Hydroxy-3-phenoxy-benzenacetonitrile	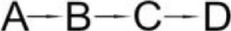	Song et al., 2015
		(C) 3-Phenoxybenzaldehyde		
		(D) 3-Phenoxybenzoic acid		
Fenvalerate (A)	*Cladosporium* sp. HU	(B) α-Hydroxy-3-phenoxy-benzenacetonitrile	No data	Chen et al., 2011a
		(C) 3-Phenoxybenzaldehyde		
Permethrin (A)	*Pseudomonas fluorescens* (SM-1)	(B) 3-Phenoxybenzyl alcohol (C) 3-(2,2-dichloroethenyl)-2,2-dimethyl cyclopropanecarboxylate (D) 3-Phenoxybenzoic acid (E) 4-Hydroxy-3-phenoxybenzoic acid	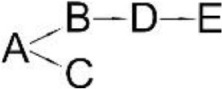	Maloney et al., 1988
	*Achromobacter* sp. (SM-2)			
	*Bacillus cereus* (SM-3)			

The most detailed degradation pathway of fenpropathrin was published for *O. tritici* pyd-1 (Wang et al., 2011). Strain pyd-1 initially hydrolyzed fenpropathrin to produce cyano-3-phenoxybenzylalcohol and 2,2,3,3-tetramethyl cyclopropanecarboxylic acid. The first product was converted into 3-PBA, which was further transformed into 3-phenoxybenzoic acid (Table 6). The cleavage of this metabolite resulted in the formation of 4-hydroxy-3-phenoxybenzoic acid and protocatechuate. The first metabolite was transformed into *p*-hydroquinone, which was subsequently converted into 3-oxoadipate *via* 1,2,4-benzenetriol and maleylacetate (Wang et al., 2011). During the degradation of fenpropathrin by *Bacillus* sp. DG-02 (Chen et al., 2014), *B. cereus* ZH-3 (Liu et al., 2015) and *Pseudomonas aeruginosa* JQ-41 (Song et al., 2015), another metabolite, i.e., α-hydroxy-3-phenoxy-benzenacetonitrile, was detected. All of the bacterial strains transformed it into 3-PBA (Table 6).

The degradation pathway of cypermethrin was studied in some strains from the *Bacillus* genera, *Micrococcus* sp. (Tallur et al., 2008), *S. aureus* (Chen et al., 2012c), *Catellibacterium* sp. (Zhao et al., 2013) and consortium that consisted of *B. licheniformis* B-1 and *Sphingomonas* sp. SC-1 (Liu et al., 2014) as well as *B. cereus* ZH-3 and *S. aureus* HP-S-01 (Chen et al., 2012b; Table 6). The metabolites that were detected during cypermethrin degradation by *Bacillus* sp. ISTDS2 and *Bacillus* sp. SG2, *B. subtilis* BSF01 were α-hydroxy-3-phenoxy-benzenacetonitrile and 3-(2,2-dichloroethenyl)-2,2-dimethyl cyclopropanecarboxylate. The first product was then metabolized into 3-phenoxybenzenaldehyde (*Bacillus* sp. SG2 and *B. subtilis* BSF01) and 3-phenoxybenzoic acid (*Bacillus* sp. ISTDS2) (Table 6). Other intermediates were found during the degradation of cypermethrin by *Micrococcus* sp. CPN 1 (Tallur et al., 2008). In this case, cyano-3-phenoxybenzyl alcohol and 3-(2,2-dichlorovinyl)-2,2-dimethylcyclopropanecarboxylate were the main products of cypermethrin hydrolysis (Table 6). The first metabolite was transformed into cyano-phenoxybenzaldehyde and subsequently oxidized into 3-phenoxybenzoic acid. Its cleavage yielded phenol and protocatechuic. Another intermediate, i.e., 4-phenoxyphenol-2,2-dimethyl-propiophenone, was found in a medium inoculated with *B. cereus* ZH-3 and *S. aureus* HP-S-01 (Chen et al., 2012b).

### Factors that affect the degradation of pyrethroids in liquid media and soils

Many studies have revealed that the biodegradation process of pyrethroids in liquid media and soils is strongly dependent on many factors such as temperature, pH, nutrients, inoculum size, moisture, organic matter content, the initial pesticide concentration, and additional carbon sources as well as the properties of the bacterial or fungal strains (Saikia and Gopal, 2004; Zhang et al., 2010; Zhao et al., 2013; Cycoń et al., 2014; Chen et al., 2015; Song et al., 2015; Akbar et al., 2015a).

It has been demonstrated that the pyrethroid concentration has an important effect on their degradation rate in liquid media and therefore, pyrethroid-degrading microorganisms were studied in relation to their tolerance to a wide range of pyrethroid concentrations. In general, the higher the pyrethroid concentration used, the lower rate of degradation with or without a lag phase. For example, Chen et al. (2015) demonstrated that *Bacillus thuringiensis* strain ZS-19 degraded cyhalothrin at a concentration as high as 800 mg/L and no lag phase was observed. However, the authors showed that when the initial cyhalothrin concentration was increased to 200, 400, 600, and 800 mg/L, the degradation rates reached about 95, 87, 84, and 82% after 72 h, respectively. In another study, Xiao et al. (2015) also demonstrated the influence of pyrethroid concentration on the rate of its degradation by *B. subtilis* strain BSF01. As was revealed by the authors, about 94, 89, and 85% of cypermethrin at concentrations of 25, 50, and 100 mg/L were degraded within seven days, respectively. Moreover, approximately 80% of degradation was achieved at concentrations of 200, 300, and 400 mg/L with a longer lag phase observed. Zhao et al. (2013) also observed a prolonged lag phase (more than 2 days) when cypermethrin was applied at higher concentrations. In this study, about 90% of the cypermethrin (50, 100, and 200 mg/L) was degraded by *Catellibacterium* sp. CC-5 within 7 days, whereas, at 500 and 600 mg/L, only 68 and 56% degradation was achieved. Similarly, *C. pelliculosa* strain ZS-02, which was isolated by Chen et al. (2013), was capable of degrading bifenthrin up to a concentration as high as 600 mg/L; however, the lag phase was extended at higher bifenthrin concentrations. 87.6 and 81.4% of the initial dose of bifenthrin were degraded by strain ZS-02 at concentrations of 500 and 600 mg/L, respectively, whereas at lower concentrations, the degradation rates reached values from 91 to 97% after 5 days of incubation. The decrease in the specific pyrethroid degradation rate with an increase in the initial pyrethroid concentration implies that some pyrethroids act as an inhibitor to microorganism. This might be due to the fact that microbial growth starts slowly and requires an acclimation period before enhanced degradation occurs and the longer lag phase at higher concentrations might be because a greater number of organisms are needed to initiate the fast degradation of pyrethroids (Chen et al., 2012a, 2015; Zhao et al., 2013; Cycoń et al., 2014).

Another important environmental factor that affects the biodegradation of pyrethroids in liquid media is supplementation with an additional source of carbon, which results in an acceleration or reduction of the biodegradation rates. The first effect was observed by Chen et al. (2011b) who revealed that the degradation of β-cypermethrin by *O. lupini* DG-S-01 was significantly enhanced in the presence of glucose, beef extract and yeast extract and reached about 85, 90, and 87% within 5 days of incubation, respectively, while in the same period, the disappearance rate of pyrethroid in a liquid medium without a carbon source reached about 80%. Accelerated degradation in the presence of an additional source of carbon has also been observed for *Cladosporium* sp. HU (Chen et al., 2011a), *S. aureus* HP-S-01 (Chen et al., 2011d) and *Bacillus amyloliquefaciens* AP01 (Lee et al., 2016), utilizing fenvalerate, deltamethrin, and cypermethrin, respectively. These results indicate that co-metabolism of an easily biodegradable substrate significantly increases the growth rate of pesticide-degrading microorganism and consequently enhance the efficiency of the removal of pyrethroids in liquid media. In contrast, Tiwary and Dubey (2016) showed that glucose (1%), fructose (1%), and glycerol (0.5%) significantly decreased the biotransformation of cypermethrin by *Bacillus* sp. strain AKD1 and that the half-life of cypermethrin increased 0.5-fold, 0.5- and 0.4-fold, indicating an inhibitory effect of added compounds on the pesticide degradation. This inhibitory effect may be related with strongly decrease in medium pH as a result of additional carbon sources metabolism. Chen et al. (2015) also demonstrated that *B. thuringiensis* strain ZS-19, when inoculated into an organic medium, degraded only 86% of cyhalothrin (100 mg/L) within 72 h of incubation, whereas 100% of the cyhalothrin was removed in the same period when the strain ZS-19 was cultured in a mineral medium.

As has been shown, an extremely important factor that affects the effectiveness of the degradation of pyrethroids is pH; however, most biodegradation studies have been performed in liquid media under neutral conditions (Table 3). The effects of initial pH on the growth of *Streptomyces* HU-S-01 and the degradation of cypermethrin were studied by Chen et al. (2012c). The results indicated that the adaptive pH value for HU-S-01 growth was between 6 and 9. A higher level of the degradation of cypermethrin was observed between pH 7 and 8 with the optimum value of pH 7.5. In contrast, insignificant degradation was observed at pH values of 5.5 and 10.0. In another study, Zhao et al. (2013) showed that pH also had a strong impact on cypermethrin biodegradation. The most rapid removal of cypermethrin by *Catellibacterium* strain CC-5 occurred at a pH of 7, followed by 8 and then 6, with removal rates of 97, 84, and 78%, respectively, after 7 days of incubation. When the pH was lower than 6 or higher than 8, strain CC-5 was inactive and only a 55 and 50% degradation was achieved, respectively (Zhao et al., 2013). In addition, the degradation of β-cypermethrin by *Ochrobactrum* DG-S-01 was also affected by different pH conditions (Chen et al., 2011b). The results indicated that strain DG-S-01 was capable of degrading β-cypermethrin rapidly over a wide range of pH. The authors demonstrated that 56.8, 63.4, 89.8, 83.0 and 79.4% of degradation rates were achieved at pH 5, 6, 7, 8, and 9 within 5 days, respectively. The value of pH was also found to be a critical factor that determines the rate of the degradation of other pyrethroids by bacterial or fungal strains. Chen et al. (2011c) showed that the *Stenotrophomonas* strain ZS-S-01 was capable of degrading fenvalerate at pH 5–9. However, with increasing pH value the higher degradation rate was observed. Nearly 50% and more than 80% degradation of fenvalerate were observed in acidic and neutral conditions, respectively, within 5 days of incubation. The results of studies by Chen et al. (2011d) and Zhang et al. (2016) also demonstrated that the degradation of another pyrethroid, i.e., deltamethrin, by microorganisms was slower under acidic conditions than in neutral and alkaline pH. In general, neutral and alkaline conditions increased the rate of pyrethroid degradation while at a lower pH, this process was slower. This may be due to the fact that acidic conditions increase the stability of pyrethroids and their resistance to microbial degradation (Singh et al., 2006; Anwar et al., 2009; Cycoń et al., 2009; Zhao et al., 2013; Chen et al., 2015).

Another important environmental factor that affects the degradation of pyrethroids is temperature. The results of a study by Lin et al. (2011) revealed that *Streptomyces* sp. strain was able to grow well in a temperature range of 20–30°C with the optimum temperature of 26–28°C but could not tolerate temperatures higher than 34°C. The trend in cypermethrin degradation was consistent with the growth of strain HU-S-01. In a temperature range of 26–28°C, strain HU-S-01 degraded about 90% of the cypermethrin within 24 h but the degradation was very low (10%) at temperatures higher than 34°C (Lin et al., 2011). In another study, Zhao et al. (2013) demonstrated that at an initial cypermethrin concentration of 100 mg/L, *Catellibacterium* sp. CC-5 degraded 77, 89, 97, 95, and 74% of the insecticide at 20, 25, 30, 35, and 40°C, respectively, within 7 days. The greatest growth of strain CC-5 and the degradation rate of cypermethrin were observed at 30–35°C, whereas temperatures above 35°C resulted in a drastic reduction in the rate of the cypermethrin degradation (Zhao et al., 2013). Chen et al. (2011c) also revealed that temperature had significant effect on the degradation of fenvalerate by *Stenotrophomonas* sp. ZS-S-01. The results showed that strain ZS-S-01 grew well in temperatures ranging from 25° to 35°C and that the percentages of fenvalerate removal were more than 75%. In addition, strain ZS-S-01 was inactive when the temperature was lower than 25°C or higher than 35°C and only about 47 and 57% degradation was achieved (Chen et al., 2011c). Similarly, the results of studies by Chen et al. (2011b) and Zhang et al. (2016) demonstrated that the degradation of pyrethroids by microorganisms was slower at both high and low temperatures.

The rate of pyrethroid degradation may be also limited by the metabolites that arise from the degradation of parental compounds. In many cases, one of the main pyrethroid metabolites known for its toxicity is 3-phenoxybenzaldehyde (Wang et al., 2011; Chen et al., 2011a, 2014; Song et al., 2015; Xiao et al., 2015; Bhatt et al., 2016). It has been reported to be an antimicrobial compound and therefore could prevent the proliferation of bacteria, which resulted in the incomplete degradation of parental compounds. This phenomenon was observed during the degradation of pyrethroids by many authors (Khan et al., 1988; Xu et al., 2007; Xia et al., 2008; Chen et al., 2011b; Cycoń et al., 2014).

Many studies have revealed that the success of the remediation of pyrethroid-contaminated soils using bioaugmentation technology is strongly dependent on many environmental factors. Among the biotic factors, the most important seem to be the interactions between autochthonous and inoculated microorganisms such as predation and the competition for nutrients and niches. However, it is thought that the most important factor that influences the success of bioaugmentation is the ability of inoculants to survive in a contaminated environment (Duquenne et al., 1996; Karpouzas and Walker, 2000; Chen et al., 2012c; Cycoń et al., 2014). Another reason that bioaugmentation fails may be the loss of the degradative capabilities of the inoculants and/or the inhibition of their growth by the toxic intermediates that occur during the degradation of the parental compounds (Xu et al., 2007; Xia et al., 2008; Cycoń et al., 2014; Akbar et al., 2015b). To counteract these problems, alternative successive bioaugmentation that relies on the repeated inoculation of soil depending on the progress of the removal of contaminants as well as the use of immobilized microorganisms on various carrier materials are recommended. The immobilization of cells protects them against adverse environmental and chemical conditions and prolongs the activity and survival of the introduced microorganisms as well (Colla et al., 2014; Sáez et al., 2014; Tallur et al., 2015).

As has been shown in studies on the bioremediation of pyrethroid-contaminated soils, the use of bacterial or fungal strains that are capable of degrading pyrethroids as an inoculum at a level of 10^6^–10^10^ cfu/g or spores/g soil resulted in the accelerated degradation of insecticides and shortening their half-life values compared to the control soils with naturally occurring microorganisms in all cases (Liu et al., 2014; Chen et al., 2015; Akbar et al., 2015a; Zhang et al., 2016). However, it has also been observed in the case of biodegradation of other pesticides that when lower inoculum densities were used, only a small part of the introduced bacteria survived the initial competition and participated in pesticide degradation (Ramadan et al., 1990; Karpouzas and Walker, 2000). A higher initial inoculum can compensate for the decline in the initial population and the survivors can multiply and degrade pollutants (Comeau et al., 1993; Duquenne et al., 1996; Karpouzas and Walker, 2000). Some authors have also observed an initial lag phase in pyrethroid degradation after the introduction of microorganisms at an even higher level (Cycoń et al., 2014). This phenomenon may be related to the necessity for the bacteria to adapt to the presence of the pyrethroid and the adaptation of the introduced strain to soil conditions as well as the proliferation of indigenous bacteria that can use the pyrethroid as an additional source of carbon and energy. A retardation of pesticide dissipation may also be related to the competition of the inoculated strains with other microorganisms or antagonistic inhibition *via* substances that are synthesized by the indigenous microorganisms. Moreover, a relatively high pyrethroid concentration may also be an important factor that affects microbial growth (Karpouzas and Walker, 2000; Singh et al., 2006; Chen et al., 2014; Cycoń et al., 2014). Despite these limitations, many studies have shown that the introduction of pyrethroid-degrading microorganisms into soil results in the accelerated degradation of pyrethroids (Table 4). The observed enhancement of the elimination of pyrethroids and other pesticides in many studies can be explained by the fact that the introduced bacterial or fungal strains increased the catabolic potential of the soils and moreover, that autochthonous soil microorganisms had the synergistic ability to degrade certain pesticides (Singh et al., 2006; Chen et al., 2012d; Cycoń et al., 2013; Sundaram et al., 2013; Zhang et al., 2016).

It has been shown that simultaneous biostimulation and bioaugmentation appears to be the most effective in the removal of some pesticides from contaminated soils (Kataoka et al., 2011; Guo et al., 2014). In the case of pyrethroids, Chen et al. (2012c) reported that the supplementation of sucrose to β-cypermethrin-contaminated soil inoculated with *S. aureus* HP-S-01 slightly enhanced the disappearance of this compound, which indicates that co-metabolism with another carbon source could possibly improve the bioremediation efficiency of these compounds by inoculating strain HP-S-01 into soils. As was revealed by the authors, the disappearance process with sucrose was stimulated and fast during the incubation period (0–10 days), about 89 and 86% of the initial dose of β-cypermethrin was eliminated in non-sterilized and sterilized soils, respectively, while the disappearance rate of β-cypermethrin in soils without sucrose was lower and reached nearly 88 and 81%, respectively, for non-sterilized and sterilized soils in the same period. The half-life of β-cypermethrin in non-sterilized and sterilized soils in the presence of sucrose decreased in comparison with the disappearance period in soils without an additional carbon source (Chen et al., 2012c).

## Conclusions and future perspectives

The confirmed negative effect of pyrethroids on the water and terrestrial organisms force us to development of technologies that guarantee the safe, efficient and economical way of pesticide removal from the contaminated environments. Among the different methods that have been proposed the use of pesticide-degrading microorganisms with effective hydrolyzing enzyme is thought to be the most promising. The data presented in this review highlights the great potential of many bacterial and fungal strains to degrade a wide range of pyrethroids. The most useful for bioremediation process are microorganisms capable of degrading many pyrethroids. To overcome problem related to the formation and further accumulation of toxic metabolites during pesticide degradation the consortia consisting of various bacteria or bacteria and fungi may be applied. Since the members of consortia differ in the ability to degrade pyrethroid and their intermediate the toxic products may serve as a substrate for given microorganism of consortium. However, there are only few studies on such cooperative degradation activities. In order to achieve the higher efficiency in pesticide removal the studies on the relation between members of such consortium, interaction with pesticides and environmental conditions should be carried out. The efforts should also be put on screening of bacteria that will be able to work under a wide range of soil environmental factors such pH, temperature, salinity, heavy metals and nutrient availability. Detailed knowledge about the pathways of pesticide degradation, the enzymes involved in catalytic reactions and the genes encoding the key enzyme will allow designing new alternatives in the remediation technology. An example of such innovative technique is the enzyme-catalyzed degradation of pesticides that may be more efficiently than existing chemical methods. The progress in the use of microorganisms in cleaning up of polluted soils may be also achieved thanks to application of new analytical and molecular tools. Genetic engineering techniques enable to the modification of targeted genes that encode the enzymes in the metabolic pathways and the construct of microorganisms with higher degradation potential or/and able to degrade other pesticides. Moreover, genomic and proteomic techniques, applied to environmentally important microorganisms, may reveal key features of biodegradation pathways and the ability of pesticide degraders to adapt to changing environmental conditions. Since many abiotic factors such as temperature, pH, nutrients, moisture, organic matter content, and the initial pesticide concentration, as well as additional carbon sources affect the biodegradation process and pyrethroid-degrading microorganisms, further studies related to the interactions of these microorganisms with the soil environment are still needed before their application in field-scale bioremediation.

## Author contributions

MC conceived and designed content of the paper and prepared the figures and tables; MC, ZP wrote the paper.

### Conflict of interest statement

The authors declare that the research was conducted in the absence of any commercial or financial relationships that could be construed as a potential conflict of interest.
